# Diversity of Natural Product Biosynthetic Genes in the Microbiome of the Deep Sea Sponges *Inflatella pellicula, Poecillastra compressa*, and *Stelletta normani*

**DOI:** 10.3389/fmicb.2016.01027

**Published:** 2016-06-29

**Authors:** Erik Borchert, Stephen A. Jackson, Fergal O’Gara, Alan D. W. Dobson

**Affiliations:** ^1^School of Microbiology, University College Cork, National University of IrelandCork, Ireland; ^2^Biomerit Research Centre, University College Cork, National University of IrelandCork, Ireland; ^3^School of Biomedical Sciences, Curtin Health Innovation Research Institute, Curtin UniversityPerth, WA, Australia; ^4^Environmental Research Institute, University College Cork, National University of IrelandCork, Ireland

**Keywords:** pyrosequencing, deep sea sponges, ketosynthase domain, adenylation domain, microbiome

## Abstract

Three different deep sea sponge species, *Inflatella pellicula, Poecillastra compressa*, and *Stelletta normani* comprising seven individual samples, retrieved from depths of 760–2900 m below sea level, were investigated using 454 pyrosequencing for their secondary metabolomic potential targeting adenylation domain and ketosynthase domain sequences. The data obtained suggest a diverse microbial origin of nonribosomal peptide synthetases and polyketide synthase fragments that in part correlates with their respective microbial community structures that were previously described and reveals an untapped source of potential novelty. The sequences, especially the ketosynthase fragments, display extensive clade formations which are clearly distinct from sequences hosted in public databases, therefore highlighting the potential of the microbiome of these deep sea sponges to produce potentially novel small-molecule chemistry. Furthermore, sequence similarities to gene clusters known to be involved in the production of many classes of antibiotics and toxins including lipopeptides, glycopeptides, macrolides, and hepatotoxins were also identified.

## Introduction

Marine sponges (Porifera) are important members of marine benthic communities in our oceans, and they continue to attract attention due to their remarkably diverse bacterial, archaeal, and eukaryotic microbial community structures (Webster and Taylor, 2012), and their importance as a source of novel natural products. Many of the sponge-microbial associates are symbionts involved in nutrient cycling and may also play a role in the sponge’s chemical defense mechanisms (Taylor et al., 2007; Bell, 2008; Webster et al., 2010; Hentschel et al., 2012). Sponges are typically sessile filter feeders, filtering large quantities of seawater which contain microbes and viruses that are potentially harmful to the sponge. Thus, the ability of members of their microbial communities to produce secondary metabolites with the potential to augment the sponges’ own chemical defense mechanisms is likely to be advantageous. Sponges are one of the oldest extant metazoans on earth and appear to be obligatorily associated with their bacterial endosymbiotic communities. It is reasonable to expect divergent evolution of ancestral genes among these endosymbionts to the extent that the resulting gene products are likely to be significantly different to those of a terrestrial origin. This is likely to be particularly true of the endosymbionts of deep sea sponges which have been exposed to extremes of temperature, salinity, and pressure for many millions of years. The adaptation of sponge endosymbionts to these extreme conditions can be expected to also have been facilitated by increased horizontal gene transfer frequencies that are known to be high amongst marine microbial communities, resulting in increases in the genomic flexibility within these bacterial populations (Penn et al., 2009; Sobecky and Hazen, 2009; McDaniel et al., 2010).

Numerous studies have been undertaken to date to investigate the microbial ecology and the biological potential of marine shallow water habitats (Aylward et al., 2015). In marked contrast even though our oceans have a mean depth of 3800 m, with 50% being below 3000 m deep, deep sea marine environments have only rarely been explored with respect to their potential to genetically encode secondary metabolites of clinical or industrial utility (Ramirez-Llodra et al., 2010). This lack of exploration is most likely due to the technical difficulties and costs associated with sampling at lower depths, with only 5% of the ‘deep sea’ having to date been explored with remote instrumentation (Ramirez-Llodra et al., 2010). Therefore, it can be assumed that to date we have only ‘scratched the surface’ of the true biotechnological potential of our oceans, particularly the deep sea.

The identification of novel bioactive compounds and the metabolic potential of microbial communities from various terrestrial or marine habitats have mostly been investigated using a variety of different approaches including direct chemical extraction methods, enhancing cultivability of microorganisms (Sipkema et al., 2011), and testing of isolated microorganisms (Gurgui and Piel, 2010). Novel natural products from the marine environment include, for example, new antimicrobial agents (Jang et al., 2013), novel bioactive compounds (Reen et al., 2015), antifouling agents (Fusetani, 2011), and various enzymes of industrial interest (Satpute et al., 2010; Kennedy et al., 2011; Jackson et al., 2015). However, the overall diversity of the secondary metabolite biosynthetic potential present within these environments is difficult to assess given that the majority of bacteria are not readily cultured using currently available microbiological methods (Uria and Piel, 2009).

Polyketide synthase (PKS) and nonribosomal peptide synthetase (NRPS) gene clusters encode for modular arrangements of different enzymes that are able to extend, modify, connect, and alter a variety of substrates to produce unique compounds with specific enzymatic, chemical, or antimicrobial properties (Hertweck, 2009; Khosla, 2009; Helfrich et al., 2014). Each PKS or NRPS gene cluster produces a specific secondary metabolite and the presence of diversity in these gene clusters is indicative of diverse secondary metabolism products. The conserved nature of PKS and NRPS allows the design of degenerate primers to target specific domains which these gene clusters have in common, such as ketosynthase domains (KSs) in PKS or adenylation and condensation domains in NRPS clusters (Reddy et al., 2012; Woodhouse et al., 2013; Charlop-Powers et al., 2014). To assess these clusters and to help overcome the problems associated with culture-dependent approaches, efforts have been focused on the analysis of community DNA isolated directly from the environment in question, which can provide a means of exploring their secondary metabolic potential (Trindade-Silva et al., 2013; Woodhouse et al., 2013). Nonetheless, to date, only a few studies have been published which have investigated the secondary metabolic potential of a mixed microbial community using next-generation sequencing (NGS) technologies. The resultant sequencing depths have the potential to reveal the entire secondary metabolomic potential of a microbial cohort, something not achievable prior to the advent of NGS. Previous NGS studies targeting secondary metabolism genes have focused on soils (Reddy et al., 2012; Charlop-Powers et al., 2014) and marine sponges (Woodhouse et al., 2013). NGS technologies have to date been primarily used to study microbial abundance via 16S rRNA gene sequencing (Sogin et al., 2006). In contrast, clone libraries, functional metagenomic libraries, and comparable techniques have been used to target secondary metabolite gene clusters to estimate the metabolic potential of a given microbial community (Rocha-Martin et al., 2014). Reddy et al. (2012) investigated three geographically distinct soil samples and found comparably similar distribution of major bacterial phyla in those soils using 16S rRNA gene analysis, but almost completely distinct sets of secondary metabolite biosynthetic gene sequences. In that study, they investigated the presence of specific parts of PKS, NRPS, and PKS/NRPS hybrid clusters, namely the KS of Type I PKS and the adenylation domain (AD) of NRPS clusters. The primers they used were designed to amplify conserved regions of these domains, including the catalytic active site and yielded a PCR product of approximately 795 and 760 bp for AD and KS domains, respectively (Ayuso-Sacido and Genilloud, 2005; Schirmer et al., 2005), which correlates with the expected average size of 454 pyrosequencing reads.

We have previously investigated the microbial diversity of the deep sea sponges *Inflatella pellicula*, *Poecillastra compressa*, and *Stelletta normani* by 16S rRNA gene pyrosequencing and found that they contained diverse bacteria and archaea, with *I. pellicula* in particular being dominated by archaea (Jackson et al., 2013; Kennedy et al., 2014). Here, we investigate the potential for secondary metabolite production of the microbiome of these deep sea sponges to produce novel natural products, utilizing 454 pyrosequencing, targeting PKS and NRPS gene clusters, using the aforementioned Reddy et al. (2012) PCR primer sets. We report that the microbial communities associated with these deep sea sponges do indeed harbor a wide variety of these genes. The results clearly show relatedness to genes that are involved in the synthesis of known classes of bioactive compounds, for example, lipopeptides, glycopeptides, macrolides, and hepatotoxins. However, and importantly, there is also a large proportion of comparably different sequences which are only distantly related to domains from known Type I PKS and NRPS sequences.

## Materials and Methods

### Sample Collection

Sponge samples (*n* = 7) of the species *S. normani*, *I. pellicula*, and *P. compressa* were collected in Irish territorial waters off the west coast of Ireland using the remotely operated vehicle (ROV) Holland I during the Biodiscovery cruises 2010 (2 × *I. pellicula*, 1 × *S. normani*, and 1 × *P. compressa*) and 2013 (2 × *S. normani* and 1 × *P. compressa*) aboard the R.V. Celtic Explorer (**Table 1**). After collection, the samples were rinsed with sterile artificial seawater [3.33% (w/v) Instant Ocean, Aquarium Systems] to remove exogenous materials and stored at –80°C until further processing.

**Table 1:** Sample collection data.

Sample	ID	Latitude	Longitude	Depths (m)
*Inflatella pellicula*^a^	BD226	54.2419	–12.6938	2900
*I. pellicula*^a^	BD92	54.0015	–12.3100	748
*Stelletta normani*^a^	BD243	54.0015	–12.3100	1350
*S. normani*	BDV1267	54.0500	–12.5333	2400
*S. normani*	BDV1379	53.9861	–12.6100	760
*Poecillastra compressa*^a^	BD130	54.0633	–12.4131	1469
*P. compressa*	BDV1346	54.0500	–12.5833	1250

^a^*Samples also used in Jackson et al. (2013) and Kennedy et al. (2014) to generate 16S rRNA data.*

### Metagenomic DNA Extraction and Purification

Frozen sponge tissues of all samples were ground in a sterile mortar with a pestle under liquid nitrogen. The obtained ground tissue was suspended in lysis buffer [100 mM Tris, 100 mM EDTA, 1.5 M NaCl (w/v), 1% CTAB (w/v), 2% SDS (w/v)] in a 1:5 ratio and subsequently incubated for 2 h at 70°C (Kennedy et al., 2008). Solution was centrifuged until a clear solution was obtained. Afterward, DNA was precipitated using 0.7 volumes of isopropanol for 30 min at room temperature, followed by centrifugation at 6000 × *g* for 30 min. Supernatant was discarded, pellet was washed with 70% ethanol, centrifuged again, after supernatant removal air dried and finally resuspended in an appropriate amount of Tris–EDTA buffer (10 mM Tris, 1 mM EDTA, pH 8.0). The metagenomic DNA was then analyzed by gel electrophoresis, spectrophotometrically quantified (NanoDrop ND-1000) and stored at –20°C until usage.

### PCR Amplicon Generation

Primer design was adapted from Reddy et al. (2012). In short, each primer consists of a 454 sequencing adaptor, a unique 10 bp identifier tag to allow for sequencing different amplicons/genes in the same region of a 454 plate and degenerate target sequence to either amplify a fragment (approximately 795 bp) of a conserved region in NRPS AD or a fragment (approximately 760 bp) of a KS from Type I PKS (see Supplementary Table S1).

For the amplification of AD gene fragments from seven samples, three different PCR conditions were used. The first reaction mixture (50 μl) comprised 10 ng DNA, 0.5 μM each primer, 200 μM deoxynucleoside triphosphate (dNTP), 1 × Q5 reaction buffer (New England Biolabs), and 1 U Q5 Hot start DNA polymerase (New England Biolabs). PCR amplification conditions for mix one were 35 cycles of 98°C for 10 s, 70°C for 30 s, 72°C for 30 s, followed by a final extension at 72°C for 3 min. The second mix contained 1 × Phusion buffer (New England Biolabs), 10 ng DNA, 200 μM dNTPs, 0.5 μM each primer, and 1 U Phusion polymerase. PCR amplification from the second reaction mixture comprised 30 cycles of 98°C for 10 s, 68°C for 30 s, 72°C for 30 s, and a final extension step at 72°C for 5 min. The third mix included 1 × Failsafe buffer E (Epicentre, FailSafe PCR System) 10 ng DNA, 200 μM dNTPs, 0.5 μM each primer, and 2.5 U DreamTaq DNA polymerase (ThermoFisher Scientific). Third mix amplification was as follows: 35 cycles of 95°C for 60 s, 60°C for 60 s, 72°C for 2 min, and a final extension at 72°C for 10 min.

For the amplification of KS fragments from five samples, only one PCR mix was employed, which is similar to the third mix from the AD amplification, except that buffer E was replaced with buffer F from the FailSafe PCR system. Conditions for amplification were as follows: 35 cycles of 95°C for 40 s, 50°C for 40 s, 72°C for 75 s, and a final extension for 5 min at 72°C (Brady, 2007). All samples were used for AD amplification, but only five for KS amplification (all three *S. normani*, one *I. pellicula*, and one *P. compressa* (2010 Cruise) sample.

### Pyrosequencing and Data Processing

The amplicons were gel purified and quantified using a spectrophotometer (NanoDrop ND-1000) and a fluorometer (Qubit^TM^ Fluorometer, Invitrogen). For library preparation, amplicons generated from all 12 samples were pooled into a single sample to a final concentration of 1.26 × 10^9^ molecules/μl and pyrosequenced on 1/8th of a plate for a 454 GS-FLX+ (Macrogen Inc.) sequencing run. The resulting sequences were quality filtered by removal of low quality (mean quality score below 25), short (less than 150 bp), homopolymer (limit of 6) and ambiguous reads (read contains more than six ambiguous bases), and sorted by sample species using QIIME (Caporaso et al., 2010). MG-RAST (Meyer et al., 2008) was used to dereplicate the quality-filtered reads, resulting in deletion of 56.9% of AD and 68.6% of KS sequences, respectively. Manually constructed and publicly available reference sequence databases were used to sort/identify the quality-filtered sequences using QIIME and NaPDos (*e*-Value Cutoff of 1e^−5^ and minimum match length of 100 aa; Ziemert et al., 2012). Manually constructed reference databases were established by screening the NCBI database for primer targets and screening known secondary metabolite gene clusters for primer-binding sites and by confirming that the adjacent sequences were either KS or ADs. In this way, each reference dataset comprised 30–40 unique sequences, which were then used in QIIME to pick reference operational taxonomic units (OTUs) (pick_open_reference_otus.py) using the UCLUST algorithm (Edgar, 2010) with pre-clustering at 60% identity to the references. The resultant representative OTUs were analyzed using MEGA, iTOL (Letunic and Bork, 2007, 2011), and MG-RAST (Meyer et al., 2008). The NaPDos tool was used to compare the obtained representative KS OTUs to sequences deposited in this database and to calculate phylogenetic trees, later visualized by iTOL. Representative sequences were also checked manually by using the BLAST algorithm against the NCBI database to exclude unwanted sequences, for example, fatty-acid-production-affiliated sequences, and to verify the AD and KS domain character of the sequence reads. The data (raw reads) are deposited in the NCBI Sequence Read Archive (SRA) database under the accession number SRP070811 (BioProject PRJNA310842).

## Results

The 454 pyrosequencing resulted in 109,079 reads of which 57,993 passed quality filtering and were subsequently analyzed downstream. Of these 57,993 sequences, 2385 reads account for AD domain sequences and 55,608 reads account for KS domain sequences. Dereplication using MG-RAST (Gomez-Alvarez et al., 2009) resulted in 15,865 unique sequences, 1621 AD reads, and 14,244 KS reads, respectively. The average length of the remaining sequences after dereplication was 398 ± 205 bp (AD) and 473 ± 168 bp (KS). A breakdown of the numbers of sequences included for further analysis and the representative sequences are provided in **Table 2**. Chao1 and Shannon diversity estimates were calculated using QIIME with 3% divergence and are listed in **Table 3** for each individual sample.

**Table 2:** Breakdown of retrieved sequences after quality control and number of picked reference OTUs.

Species	No. of sequences	Average length (bp)	GC content (%)	No. of reads after dereplication	No. of rep. OTUs
*I. pellicula* AD	760	427	66.1	351	35
*P. compressa* AD	688	249	62.8	664	14
*S. normani* AD	937	485	67.8	606	31
*I. pellicula* KS	10,227	505	53.1	3125	72
*P. compressa* KS	8167	467	49.6	2514	50
*S. normani* KS	37,214	485	57.1	8605	109

**Table 3:** Chao1 and Shannon diversity indices.

Sample	Chao1	Shannon
*I. pellicula* A AD	4.0	0.87
*I. pellicula* B AD	31.0	4.89
*P. compressa* A AD	13.0	2.19
*P. compressa* B AD	1.0	0
*S. normani* A AD	12.33	2.95
*S. normani* B AD	19.3	4.16
*S. normani* C AD	8.0	2.88
*I. pellicula* B KS	76.0	5.92
*P. compressa* A KS	50.0	4.98
*S. normani* A KS	56.12	5.08
*S. normani* B KS	5.0	2.19
*S. normani* C KS	59.13	5.70

**FIGURE 1:**
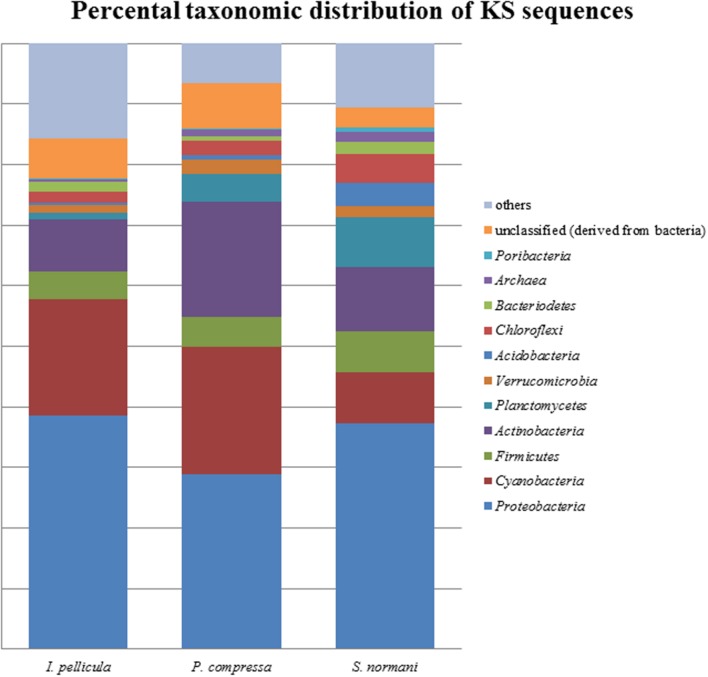
**Percental distribution of KS sequences**. Barchart based on taxonomic identification of raw reads by MG-RAST after dereplication.

**FIGURE 2:**
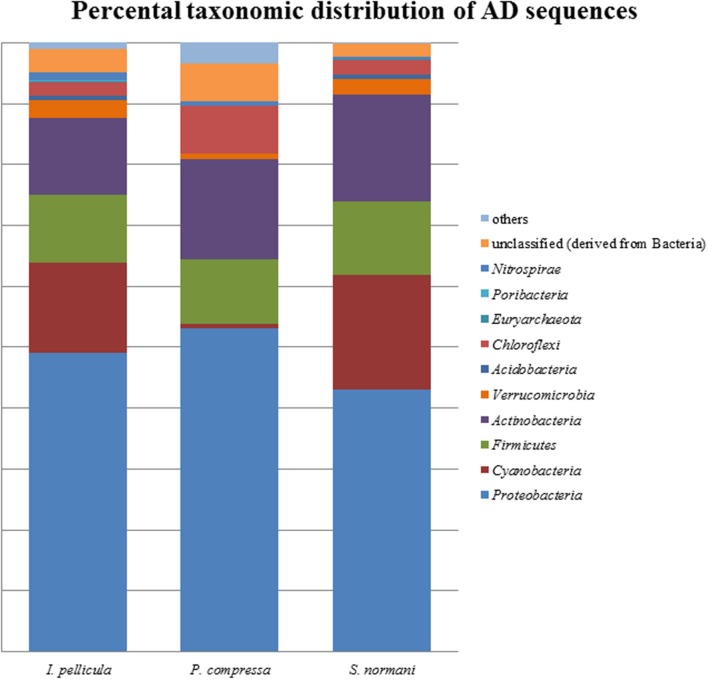
**Percental distribution of AD sequences**. Barchart based on taxonomic identification of raw reads by MG-RAST after dereplication.

The taxonomic abundances were calculated by MG-RAST after dereplication of the quality-filtered reads. The most dominant phylogenetic assignations in the AD sequences comprise *Proteobacteria, Cyanobacteria, Firmicutes, Actinobacteria, Verrucomicrobia*, and *Chloroflexi* (**Figure 1**). *Proteobacteria* account for 49% of the sequences from *I. pellicula*, 53% from *P. compressa* and for 43% from *S. normani*, and is, therefore, the most abundant phylum contributing AD sequences in all three sponge species. A difference in the abundances is observable in the amount of cyanobacterial (0.87%) and *Chloroflexi* (7.82%)-affiliated sequences in *P. compressa* in contrast to *I. pellicula* (14.7%, 2.14%) and *S. normani* (18.8%, 2.23%), respectively. The KS sequences are dominated by *Proteobacteria, Cyanobacteria, Firmicutes, Actinobacteria, Planctomycetes*, and *Verrucomicrobia* (**Figure 2**). The proteobacterial KS sequences represent 38.63, 28.94, and 37.26% of the sequences in *I. pellicula*, *P. compressa*, and *S. normani*, respectively. Observable differences are notable in the percentile distribution of *Cyanobacteria* (19.15% *I. pellicula* and 21.04% *P. compressa* in contrast to 8.34% in *S. normani*), *Actinobacteria* (8.53% in *I. pellicula* and 10.53% in *S. normani* in contrast to 19.15% in *P. compressa*), *Planctomycetes* (1% in *I. pellicula*, 4.49% in *P. compressa* and 8.18% in *S. normani*), *Firmicutes* (4.65% *I. pellicula* and 4.79% *P. compressa* and 6.92% in *S. normani*) and Verrucomicrobia (1.38% *I. pellicula*, 2.44% *P. compressa*, and 2% *S. normani*)-derived KS sequences.

### Inflatella pellicula

**FIGURE 3:**
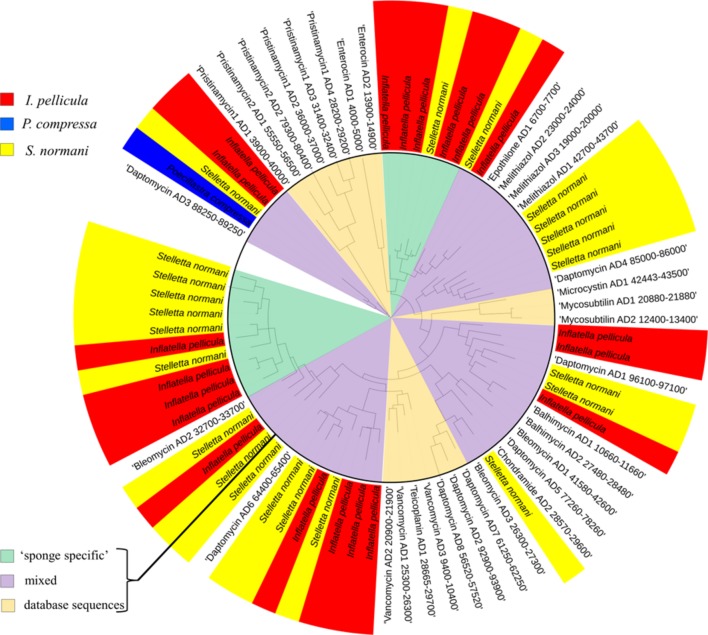
**Phylogenetic distribution of obtained reference AD sequences compared to a manually constructed reference sequence dataset**. The alignment was performed in MEGA software using CLUSTAL W (Thompson et al., 1994) for nucleotide alignment. For phylogenetic tree construction, the results were transferred to iTOL software. The sequences from *Poecillastra compressa* are blue colored, from *Inflatella pellicula* red colored, and from *Stelletta normani* yellow colored. The inner circle highlights the origin of different sequences, pale orange indicates populated only by reference sequences from the manually constructed reference dataset, green clades only comprise sponge-derived sequences and purple clades represent mixed clades.

The sponge samples from *I. pellicula* yielded 351 AD sequences and 3125 KS sequences after quality filtering and dereplication in QIIME and MG-RAST, resulting in 35 and 72 merged unique representative sequences, respectively. Of the 35 AD sequences, 18 have a length over 190 bp (up to 697 bp) and were identified as true AD sequences by BLASTX searches. The predicted taxonomic origin of these sequences is diverse with similarities to genes from species including *Clostridium* sp.*, Pseudomonas* sp.*, Sorangium cellulosum, Microcystis* sp.*, Micromonospora* sp.*, Streptomyces* sp.*, Silvibacterium bohemicum, Nostoc* sp., with *Streptomyces* sp.*, Microcystis* sp. and *S. cellulosum* being the most prominent origins (level of protein identity ranging from 40 to 60%). As can be seen from **Figure 3** (*I. pellicula* tag is colored in red) the obtained reference sequences seem to be distantly related to ADs from macrolides (Epothilone), lipopeptides (Daptomycin) and glycopeptides biosynthetic gene clusters (Vancomycin, Bleomycin, Balhimycin) and dissimilar to streptogramine (Pristinamycin), cyanoginosine (Microcystin), bacteriocin (Enterocin) or depsipetide (Chondramides) biosynthetic genes (AD domains of compounds in brackets were used to construct the reference dataset; **Figure 3**).

**FIGURE 4:**
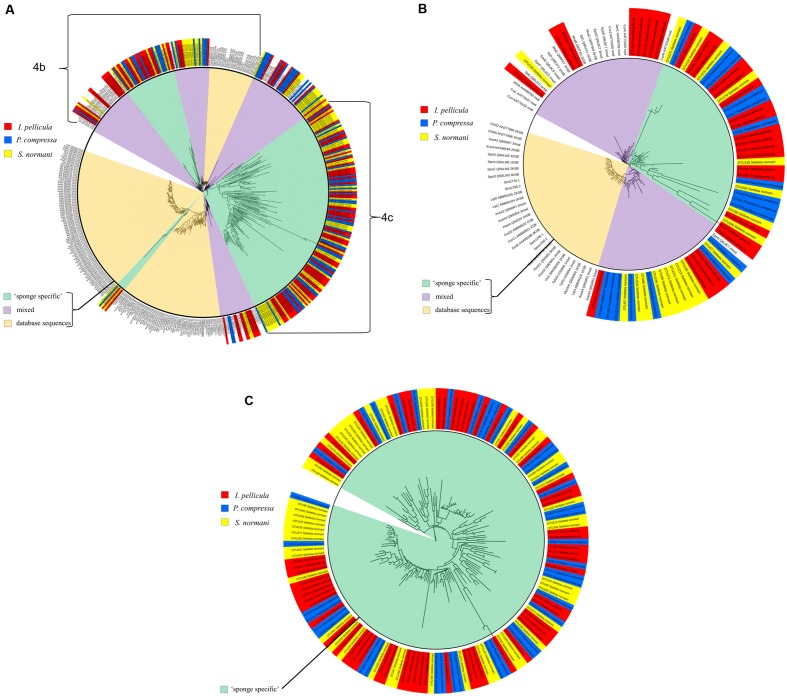
**Phylogenetic distribution of obtained reference KS sequences compared to reference sequences from NaPDos**. The KS domain detection settings were set to minimal length of 100 aa and an *e*-Value Cutoff of 1e^−5^. For phylogenetic tree construction, the results were transferred to the iTOL software. The sequences from *P. compressa* are blue colored, from *I. pellicula* red colored, and from *S. normani* yellow colored. The inner circle highlights the origin of different sequences, pale orange indicates populated only by reference sequences from the NaPDos database, green clades only comprise sponge-derived sequences (‘sponge-specific’) and purple clades represent mixed clades. **(A)** Shows the phylogenetic tree of all obtained reference KS sequences. **(B)** is a subtree of **(A)** displaying the three different kinds of observed clade formation. **(C)** Shows a large clade solely made up of obtained reference KS sequences unrelated to reference sequences from the NaPDos database.

The KS sequences were not manually checked after reference sequence picking by QIIME, but rather a second quality control step was used by analyzing the sequences with the NaPDos database. This repository consists of 96 different PKS, NRPS, and PKS/NRPS hybrid pathways with chemically characterized products. These pathways comprise 648 reference sequences for KS and condensation domains as each pathway may contain several KS or C domains (see Supplementary Table S2 for alignment scores). The putative taxonomic origin of the KS domain sequences consists of *Mycobacteria* sp.*, Cylindrospermum* sp*., Lyngbya majuscula, S. cellulosum, Paenibacillus* sp.*, Candidatus Endobugula sertula, Burkholderia* sp.*, S. aurantica, Streptomyces* sp., and many more with uncultured bacteria of marine origin and cyanobacteria being the most prominent (protein identity levels varies from 40 to 70%). Phylogenetic clustering (**Figure 4A**) of the KS domain sequences (*I. pellicula* red) resulted in clade formation (purple sector, ‘mixed’) with reference KS sequences from known lipopeptide, macrolide biosynthetic genes and a large clade of diverse sequences which were unaffiliated (green sector, ‘sponge specific’) to a reference sequence.

### Poecillastra compressa

The sponge samples from *P. compressa* yielded 664 AD sequences and 2,514 KS sequences after dereplication, resulting in 14 and 50 merged unique representative sequences, respectively. Of the 14 AD sequences, only one was found to be a true AD with a considerable length (325 bp). The remaining 13 sequences comprised short reads (120–160 bp) with similarities to elongation factors or hypothetical proteins. BLASTX search of the single AD sequence displayed a 51% protein identity to a protein from *Streptomyces* sp. and 39% identity to tyrocidine synthase 3 (tyrocidine is a cyclic decapeptide) from a *Streptomyces* sp.

Forty nine of the 50 KS domain sequences passed the second quality control step. BLASTX was used to investigate the taxonomic origin of these KS sequences, resulting in similarities to previously reported KS sequences from *L. majuscula, S. aurantica, Mycobacterium* sp.*, Chondromyces apiculatus, S. cellulosum, Streptomyces* sp*., and Nannocystis pusilla*. The majority of these KS sequences displayed most similarity to Cyanobacteria and to KS sequences from uncultured bacteria of both soil and marine origin. Clustering of the KS sequences (*P. compressa* blue colored tag in **Figure 4A**) was performed with the NaPDos reference database and yielded clade formation to KS sequences from known bioactive compounds, such as streptogramins, lipopeptides, polyethers, orthosomycin antibiotics, and macrolides. Clades were also formed which were clearly distinct from the reference sequences (**Figure 4A**); with protein identity levels ranging from 37 to 75%.

### Stelletta normani

The sponge samples from *S. normani* yielded 606 AD sequences and 8,605 KS sequences after dereplication. This resulted in 31 and 109 merged unique representative sequences, respectively, and is therefore the most diverse of the three sample species. Five of the 31 AD domain sequences were discarded due to length restrictions (shorter than 180 bp). A BLASTX search was conducted to look for protein similarities and similarities were predominately found to proteins from *Bacillus* sp*., Stigmatella aurantica, Hyella* sp*., Nostoc* sp. and *Microcystis* sp.*, Cylindrospermum* sp.*, Brevibacillus* sp.*, Streptomyces* sp.*, Planktothrix* sp*., Nitratireductor* sp.*, and Methylobacter* sp. When clustered with known AD domain sequences the obtained sequences (*S. normani* sequences tagged yellow, **Figure 3**) formed clades with genes that produce lipopeptides, glycopeptides and with sequences derived from the beta-methoxyacrylate inhibitor Melithiazol, with some sequences clustering apart from the reference sequence (**Figure 3**).

The initial reference sequence picking via QIIME resulted in 109 sequences, of which 55 passed the second quality filter step (NaPDos). BLASTX search of these sequences yielded similarities to proteins from *S. cellulosum, Amycolatopsis* sp.*, Mycobacterium* sp.*, Streptomyces* sp.*, Scytonema* sp.*, L. majuscula, Clostridium* sp.*, Candidatus Thiomargarita nelsonii* and to KS sequences from both uncultured soil and marine bacteria were observed. The KS sequences cluster with biosynthetic genes from lipopeptides, orthosomycin antibiotic, macrolides and a large cluster of diverse sequences distantly related to KS sequences from gene clusters known to produce Jamaicamides and Melithiazol (**Figure 4A**).

## Discussion

The secondary metabolomic potential of the microbiome of three different deep sea sponge species, *I. pellicula*, *P. compressa*, and *S. normani* was investigated using 454 pyrosequencing; to detect the presence of PKS and NRPS gene-cluster-associated genes, targeting AD and KS domain sequences (**Table 1**). The use of an NGS approach, circumvents the problems associated with the cultivation of bacteria from these sponges. This study supplements a previous 16S rRNA gene-based approach we had employed to study the microbial ecology of these deep sea sponges (Jackson et al., 2013; Kennedy et al., 2014). Given that NGS analysis of marine sponge metagenomes result in the generation of large datasets (**Table 2**), it is therefore important that strict quality control is employed so as not to lead to incorrect interpretation of the data. To reflect this, the number of raw reads used here has been reduced by approximately 85% in total, 46.8% after quality filtering, and 38.5% after dereplication (using default parameters in QIIME and MG-RAST; **Table 2**).

**FIGURE 5:**
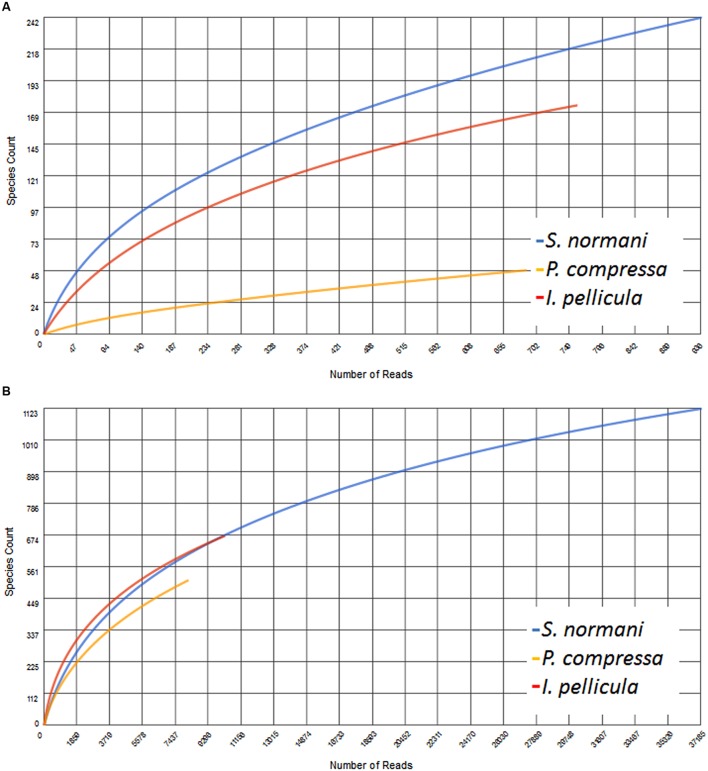
**Rarefaction curves of the obtained AD **(A)** and KS **(B)** sequences**. This figure was generated using MG-RAST where the data were compared to the Non-Redundant Multi-Source Protein Annotation Database with a minimal identity Cutoff of 60% identity to account for the observed low identity to known sequences, maximal *e*-Value Cutoff of 1e^–5^ and a minimal alignment length Cutoff of 15 aa.

The resulting number of sequences in the final analysis while clearly not representative of the entire biosynthetic potential of the sponge microbial communities is nonetheless significant in that it indicates the presence of PKS and NRPS diversity within these deep sea sponges. Rarefaction curves indicate sufficient coverage, indicated by the plateau of the curve, for only one out of three sample species (**Figure 5**). The possibility exists, as previously alluded to in Reddy et al. (2012) that the use of these degenerate primers may lead to the selective amplification of proteobacterial and actinobacterial AD and KS sequences. However, in this instance we feel that possible overrepresentation is likely to be marginal as we have previously reported that *Proteobacteria* and *Actinobacteria* account for a substantial portion of the microbial community of sponges and also of the communities in the deep sea sponges investigated here (Kennedy et al., 2014). In that study, a 16S rRNA gene-sequencing-based approach was employed to investigate the microbial communities of four deep sea sponges and of the surrounding seawater, we found that the microbial community of those sponges comprise to a large extent of *Proteobacteria* (especially γ-*Proteobacteria*), *Chloroflexi* (*S. normani*), *Actinobacteria*, and *Bacteroidetes*. The predicted taxonomic sources of the KS and AD reads presented here are in the main part, well represented in the aforementioned 16S rRNA gene dataset as well as *Firmicutes* and particularly *Cyanobacteria* (**Figures 1** and **2**), with the most prominent phyla being *Proteobacteria, Actinobacteria*, and *Cyanobacteria*. Furthermore, *Actinobacteria* or more specifically *Streptomyces* (Chater et al., 2010) and many classes of the diverse phylum of *Proteobacteria* are noted producers of potent secondary metabolites (Gerth et al., 1996; Wenzel and Müller, 2009). Though the proposed phyletic assignments of our KS and AD domain sequences are further validated by the observed similarities between the 16S rRNA gene data and the phylogenetic distribution of the KS and AD sequences, caution is required in the interpretation of these assignments. Putative taxonomic origins of functional genes are not fully reflective of the actual taxonomic source of these genes but are merely indications of sequence identity between a query sequence and its most similar sequence match. Nonetheless, the prominent occurrence of *Cyanobacteria*-affiliated sequences is puzzling as this bacterial phylum is not present in the 16S rRNA gene datasets and is not expected to be. *Cyanobacteria* rely on photosynthesis for energy generation which does not occur at depths greater than 200 m. A possible explanation for this is a high rate of horizontal gene transfer of NRPS- and PKS-cluster-affiliated sequences, which is known to frequently occur in marine sponge metagenomes. PKS and NRPS gene clusters are also known to be encoded on ‘genomic/pathogenicity islands’, that are rich in mobile genetic elements, therefore enhancing their potential transfer frequencies (Fischbach et al., 2008; Ridley et al., 2008; Ziemert et al., 2014).

The phylogenetic trees constructed from the sequences obtained for AD and KS domain fragments clearly sheds further light on the hidden biological potential of microbial populations associated with these deep sea sponges. It is evident that a portion of the sequences, in particular the KS domain sequences form their own diverse clades which are clearly distinct from KS sequences from genes encoding known bioactive compounds (**Figure 4C**). The use of the BLASTX algorithm was particularly illuminating when investigating these KS and AD sequences, given that the comparable long reads achieved with 454 pyrosequencing (up to 700 bp) allowed a more robust analysis to be performed. Many common ‘hits’ are similar to sequences of marine origin like KS sequences from uncultured bacteria identified from shallow water sponges or to *Mycobacterium marinum*, *Cyanobacteria*, and *Streptomyces* sp. Furthermore, it is worth mentioning the occasional appearance of KS domain ‘hits’ with sequences from *S. cellulosum* a myxobacterium inhabiting soil environments and the producer of Epothilone (Gerth et al., 1996). Other sequences showed similarities to genes from genera which are known to produce Hectochlorin, Jamaicamides, Gulmirecins (Schieferdecker et al., 2014), and Nostophycin (Fewer et al., 2011) among others. Hectochlorin was first isolated from the marine cyanobacterium *L. majuscula* and is a product of a mixed PKS/NRPS pathway and displays potent antifungal and cytotoxic properties (Ramaswamy et al., 2007). The Jamaicamides are lipopeptides which are also of mixed PKS/NRPS origin. They are produced by the marine cyanobacterium *L. majuscula* and display sodium channel blocking capabilities (Edwards et al., 2004). The origin of the AD domain fragments is also quite diverse with the closest ‘hits’ being to AD genes from *Brevibacillus*, *Streptomyces, Pseudomonas, Nostoc*, and *Clostridium* spp. Actual ‘hits’ with known bioactive compounds for AD sequences comprise similarities to the AD domain from Simocyclinone, an angucycline antibiotic with topoisomerase inhibitory activity (Flatman et al., 2005) and an AD domain from Microcystin which is an hepatotoxin produced by Cyanobacteria (Dawson, 1998). Furthermore, comparatively few AD domain fragments (compared to KS sequences) were retrieved from the data (2,385 before and 1,621 sequences after quality filtering) (**Table 2**), which may be due to a low abundance of this sequence type in deep sea sponges. The KS and AD domain fragment sequences can be distinguished by either clustering with reference sequences or by forming their own clades, which are only very distantly related to the database sequences used for comparison (**Figures 3** and **4A**). This is particularly true in the case of the KS sequences which make up a clade of sequences which are clearly distinct from KS sequences from genes involved in the synthesis of known bioactive compounds (**Figure 4C**). These clades are very diverse, as is evident from the individual branch lengths in the phylogenetic tree (**Figures 4A,C**). Furthermore, the KS and AD sequences show similarities to genes linked to the production of a broad range of antibiotics and toxins of different groups. These include lipopeptides, glycopeptides, macrolides, streptogramins, depsipepdtides, cyanoginosines, bacteriocins, and hepatotoxins. Thus, while sequence similarity searches and sequence cladograms indicate degrees of similarity with known PKS and NRPS gene fragments, degrees of novelty or divergence are also very obvious (**Figures 4B,C**). In particular, the KS and AD gene fragments which have been identified here form clades which are clearly distinct from those of known antibiotic-related gene clusters. This indicates that potential novel biodiversity with respect to marine natural products is likely to be present in these deep sea sponge microbiomes.

## Conclusion

In conclusion, this study reveals that PKS- and NRPS-affiliated domains are prevalent among the genomes of the members of the microbial communities of these deep sea sponges, which may potentially also be from symbiotic members of the community and therefore be sponge specific. Nonetheless further research needs to be performed to allocate the biological potential identified here to whole gene clusters and possible gene products. The exploitation of this potential may however be difficult to achieve, particularly bearing in mind the difficulties in obtaining samples from these depths and the sample size requirements involved. However given the potential biodiversity that we report here, with respect to natural product biosynthetic genes, such difficulties may be worth overcoming, particularly given the ongoing need for novel bioactive polyketides and nonribosomal peptides.

## Author Contributions

SJ and AD conceived the study. EB performed the experimental work. EB and SJ performed the data analysis. EB, SJ, FG, and AD wrote the paper.

## Conflict of Interest Statement

The authors declare that the research was conducted in the absence of any commercial or financial relationships that could be construed as a potential conflict of interest.
