# Supplementary material for: Diversity of Natural Product Biosynthetic Genes in the Microbiome of the Deep Sea Sponges Inflatella pellicula, Poecillastra compressa, and Stelletta normani
Source: Front Microbiol. 2016 Jun 29;7:1027. doi: 10.3389/fmicb.2016.01027 (PMC4925706; doi:10.3389/fmicb.2016.01027)
Supplement: Supplementary file 1 [file Table_1.DOC]

Supplementary Material

**Diversity of natural product biosynthetic genes in the deep sea sponges *Inflatella pellicula*, *Poecillastra compressa* and *Stelletta normani***

**Erik Borchert1, Stephen A. Jackson1, Fergal O’Gara1,2,3, Alan D.W. Dobson1,4***

^1^ School of Microbiology, University College Cork, National University of Ireland, Cork, Ireland

^2^ Biomerit Research Centre, University College Cork, National University of Ireland, Cork, Ireland

^3^ School of Biomedical Sciences, Curtin Health Innovation Research Institute, Curtin University, Perth, Australia.

^4^ Environmental Research Institute, University College Cork, National University of Ireland, Cork, Ireland

*** Correspondence:** Alan D.W. Dobson, School of Microbiology, University College Cork, National University of Ireland, College Road, Cork, County Cork, T12 TN60, Ireland.

a.dobson@ucc.ie

# Supplementary Figures and Tables

## Supplementary Tables

**Supplementary Table 1. Primers used in this study**

| **AD** | Sequence | Sample |
| --- | --- | --- |
| A16A3F | CGTATCGCCTCCCTCGCGCCATCAG TCACGTACTA GCSTACSYSATSTACACSTCSGG | BD243 |
| B16A7R | CTATGCGCCTTGCCAGCCCGCTCAG TCACGTACTA SASGTCVCCSGTSCGGTAS |  |
| A19A3F | CGTATCGCCTCCCTCGCGCCATCAG TGTACTACTC GCSTACSYSATSTACACSTCSGG | BD130 |
| B19A7R | CTATGCGCCTTGCCAGCCCGCTCAG TGTACTACTC SASGTCVCCSGTSCGGTAS |  |
| A50A3F | CGTATCGCCTCCCTCGCGCCATCAG ACTAGCAGTA GCSTACSYSATSTACACSTCSGG | BD226 |
| B50A7R | CTATGCGCCTTGCCAGCCCGCTCAG ACTAGCAGTA SASGTCVCCSGTSCGGTAS |  |
| A62A3F | CGTATCGCCTCCCTCGCGCCATCAG TACGTCATCA GCSTACSYSATSTACACSTCSGG | BD92 |
| B62A7R | CTATGCGCCTTGCCAGCCCGCTCAG TACGTCATCA SASGTCVCCSGTSCGGTAS |  |
| A47A3R | CGTATCGCCTCCCTCGCGCCATCAG TGTGAGTAGT GCSTACSYSATSTACACSTCSGG | BDV1267 |
| B47A7R | CTATGCGCCTTGCCAGCCCGCTCAG TGTGAGTAGT SASGTCVCCSGTSCGGTAS |  |
| A15A3F | CGTATCGCCTCCCTCGCGCCATCAG ATACGACGTA GCSTACSYSATSTACACSTCSGG | BDV1379 |
| B15A7R | CTATGCGCCTTGCCAGCCCGCTCAG ATACGACGTA SASGTCVCCSGTSCGGTAS |  |
| A52A3F | CGTATCGCCTCCCTCGCGCCATCAG AGTATACATA GCSTACSYSATSTACACSTCSGG | BDV1346 |
| B52A7R | CTATGCGCCTTGCCAGCCCGCTCAG AGTATACATA SASGTCVCCSGTSCGGTAS |  |
| **KS** |  |  |
| A38KSiF | CGTATCGCCTCCCTCGCGCCATCAG TACACGTGAT GCIATGGAYCCICARCARMGIVT | BD243 |
| B38KSiR | CTATGCGCCTTGCCAGCCCGCTCAG TACACGTGAT GTICCIGTICCRTGISCYTCIAC |  |
| A41KSiF | CGTATCGCCTCCCTCGCGCCATCAG TAGTGTAGAT GCIATGGAYCCICARCARMGIVT | BD130 |
| B41KSiR | CTATGCGCCTTGCCAGCCCGCTCAG TAGTGTAGAT GTICCIGTICCRTGISCYTCIAC |  |
| A70KSiF | CGTATCGCCTCCCTCGCGCCATCAG TGAGTCAGTA GCIATGGAYCCICARCARMGIVT | BD92 |
| B70KSiR | CTATGCGCCTTGCCAGCCCGCTCAG TGAGTCAGTA GTICCIGTICCRTGISCYTCIAC |  |
| A60KSiF | CGTATCGCCTCCCTCGCGCCATCAG CTACGCTCTA GCIATGGAYCCICARCARMGIVT | BDV1267 |
| B60KSiR | CTATGCGCCTTGCCAGCCCGCTCAG CTACGCTCTA GTICCIGTICCRTGISCYTCIAC |  |
| A37KSiF | CGTATCGCCTCCCTCGCGCCATCAG TACACACACT GCIATGGAYCCICARCARMGIVT | BDV1379 |
| B37KSiR | CTATGCGCCTTGCCAGCCCGCTCAG TACACACACT GTICCIGTICCRTGISCYTCIAC |  |

**Supplementary Table 2. Sequence alignment output of KS sequences with NapDOs database**

| **Query id** | **Database match id** | **% id.** | **align length** | **e-value** | **pathway product** | **class** |
| --- | --- | --- | --- | --- | --- | --- |
| New.CleanUp.ReferenceOTU0_IpB.KS_8412 | StiE_Q8RJY2_1KSB | 47 | 208 | 2.00E-44 | stigmatellin | modular |
| New.ReferenceOTU0_PcA.KS_7856 | CurA_AAT70096_mod | 54 | 90 | 1.00E-32 | curacin | KS |
| New.ReferenceOTU0_PcA.KS_7856 | CurA_AAT70096_mod | 37 | 83 | 1.00E-32 | curacin | KS |
| New.CleanUp.ReferenceOTU1_PcA.KS_6826 | StiG_Q8RJY0_1KSB | 63 | 105 | 6.00E-55 | stigmatellin | modular |
| New.CleanUp.ReferenceOTU1_PcA.KS_6826 | StiG_Q8RJY0_1KSB | 68 | 68 | 6.00E-55 | stigmatellin | modular |
| New.CleanUp.ReferenceOTU103_PcA.KS_6079 | StiC_Q8RJY4_1KSB | 68 | 139 | 2.00E-49 | stigmatellin | modular |
| New.CleanUp.ReferenceOTU103_PcA.KS_6079 | StiC_Q8RJY4_1KSB | 66 | 29 | 2.00E-49 | stigmatellin | modular |
| New.CleanUp.ReferenceOTU103_PcA.KS_6079 | StiC_Q8RJY4_1KSB | 53 | 17 | 2.00E-49 | stigmatellin | modular |
| New.CleanUp.ReferenceOTU104_PcA.KS_4856 | CurL_AAT70107_mod | 75 | 135 | 8.00E-41 | curacin | modular |
| New.CleanUp.ReferenceOTU106_PcA.KS_3967 | EpoE_Q9L8C6_1mod | 58 | 203 | 4.00E-57 | epothilone | modular |
| New.CleanUp.ReferenceOTU106_PcA.KS_3967 | EpoE_Q9L8C6_1mod | 81 | 16 | 4.00E-57 | epothilone | modular |
| New.CleanUp.ReferenceOTU11_PcA.KS_8003 | CurA_AAT70096_mod | 51 | 128 | 4.00E-44 | curacin | KS |
| New.CleanUp.ReferenceOTU11_PcA.KS_8003 | CurA_AAT70096_mod | 85 | 26 | 4.00E-44 | curacin | KS |
| New.CleanUp.ReferenceOTU11_PcA.KS_8003 | CurA_AAT70096_mod | 45 | 40 | 4.00E-44 | curacin | KS |
| New.CleanUp.ReferenceOTU113_PcA.KS_4694 | CALO5_12183629_i | 57 | 197 | 1.00E-56 | calicheamicin | iterative |
| New.CleanUp.ReferenceOTU113_PcA.KS_4694 | CALO5_12183629_i | 69 | 29 | 1.00E-56 | calicheamicin | iterative |
| New.CleanUp.ReferenceOTU116_PcA.KS_4922 | MtaB_Q9RFL0_1KSB | 76 | 29 | 2.00E-13 | myxothiazol | KS1 |
| New.CleanUp.ReferenceOTU116_PcA.KS_4922 | MtaB_Q9RFL0_1KSB | 54 | 28 | 2.00E-13 | myxothiazol | KS1 |
| New.CleanUp.ReferenceOTU122_PcA.KS_6145 | MtaB_Q9RFL0_2KSB | 65 | 104 | 1.00E-55 | myxothiazol | modular |
| New.CleanUp.ReferenceOTU122_PcA.KS_6145 | MtaB_Q9RFL0_2KSB | 82 | 39 | 1.00E-55 | myxothiazol | modular |
| New.CleanUp.ReferenceOTU122_PcA.KS_6145 | MtaB_Q9RFL0_2KSB | 62 | 40 | 1.00E-55 | myxothiazol | modular |
| New.CleanUp.ReferenceOTU127_PcA.KS_6364 | StiG_Q8RJY0_1KSB | 61 | 157 | 1.00E-64 | stigmatellin | modular |
| New.CleanUp.ReferenceOTU127_PcA.KS_6364 | StiG_Q8RJY0_1KSB | 63 | 30 | 1.00E-64 | stigmatellin | modular |
| New.CleanUp.ReferenceOTU127_PcA.KS_6364 | StiG_Q8RJY0_1KSB | 65 | 31 | 1.00E-64 | stigmatellin | modular |
| New.CleanUp.ReferenceOTU131_PcA.KS_7377 | EpoE_Q9L8C6_1mod | 47 | 159 | 4.00E-30 | epothilone | modular |
| New.CleanUp.ReferenceOTU134_PcA.KS_6272 | EpoD_Q9L8C7_2mod | 47 | 232 | 5.00E-52 | epothilone | modular |
| New.CleanUp.ReferenceOTU136_PcA.KS_8120 | MxaB_Q93TX0_1KSB | 61 | 163 | 2.00E-49 | myxalamid | modular |
| New.CleanUp.ReferenceOTU136_PcA.KS_8120 | MxaB_Q93TX0_1KSB | 63 | 19 | 2.00E-49 | myxalamid | modular |
| New.CleanUp.ReferenceOTU138_PcA.KS_5049 | StiG_Q8RJY0_1KSB | 50 | 165 | 6.00E-42 | stigmatellin | modular |
| New.CleanUp.ReferenceOTU138_PcA.KS_5049 | StiG_Q8RJY0_1KSB | 59 | 39 | 6.00E-42 | stigmatellin | modular |
| New.CleanUp.ReferenceOTU139_PcA.KS_6442 | MtaB_Q9RFL0_2KSB | 54 | 89 | 4.00E-42 | myxothiazol | modular |
| New.CleanUp.ReferenceOTU139_PcA.KS_6442 | MtaB_Q9RFL0_2KSB | 59 | 56 | 4.00E-42 | myxothiazol | modular |
| New.CleanUp.ReferenceOTU139_PcA.KS_6442 | MtaB_Q9RFL0_2KSB | 46 | 35 | 4.00E-42 | myxothiazol | modular |
| New.CleanUp.ReferenceOTU153_PcA.KS_6288 | TylGIII_O33956_1mod | 57 | 75 | 1.00E-29 | tylosin | modular |
| New.CleanUp.ReferenceOTU153_PcA.KS_6288 | TylGIII_O33956_1mod | 68 | 34 | 1.00E-29 | tylosin | modular |
| New.CleanUp.ReferenceOTU19_PcA.KS_4305 | CurJ_AAT70105_mod | 63 | 181 | 3.00E-64 | curacin | modular |
| New.CleanUp.ReferenceOTU19_PcA.KS_4305 | CurJ_AAT70105_mod | 52 | 40 | 3.00E-64 | curacin | modular |
| New.CleanUp.ReferenceOTU2_PcA.KS_6820 | EpoD_Q9L8C7_4mod | 78 | 80 | 2.00E-55 | epothilone | modular |
| New.CleanUp.ReferenceOTU2_PcA.KS_6820 | EpoD_Q9L8C7_4mod | 56 | 71 | 2.00E-55 | epothilone | modular |
| New.CleanUp.ReferenceOTU2_PcA.KS_6820 | EpoD_Q9L8C7_4mod | 72 | 25 | 2.00E-55 | epothilone | modular |
| New.CleanUp.ReferenceOTU21_PcA.KS_2730 | EpoD_Q9L8C7_4mod | 61 | 177 | 7.00E-51 | epothilone | modular |
| New.CleanUp.ReferenceOTU25_PcA.KS_8098 | MycAIII_Q83WE8_1KSB | 53 | 45 | 9.00E-09 | mycinamicin | modular |
| New.CleanUp.ReferenceOTU29_PcA.KS_5493 | EpoD_Q9L8C7_4mod | 67 | 199 | 2.00E-72 | epothilone | modular |
| New.CleanUp.ReferenceOTU34_PcA.KS_7090 | StiH_Q8RJX9_1KSB | 44 | 212 | 8.00E-42 | stigmatellin | modular |
| New.CleanUp.ReferenceOTU37_PcA.KS_7809 | EpoD_Q9L8C7_3mod | 53 | 90 | 3.00E-46 | epothilone | modular |
| New.CleanUp.ReferenceOTU37_PcA.KS_7809 | EpoD_Q9L8C7_3mod | 64 | 74 | 3.00E-46 | epothilone | modular |
| New.CleanUp.ReferenceOTU40_PcA.KS_6246 | KirAII_CAN89632_5T | 46 | 168 | 1.00E-51 | kirromycin | trans |
| New.CleanUp.ReferenceOTU40_PcA.KS_6246 | KirAII_CAN89632_5T | 53 | 55 | 1.00E-51 | kirromycin | trans |
| New.CleanUp.ReferenceOTU41_PcA.KS_7883 | Stro2780_2 | 45 | 147 | 4.00E-26 | salinilactam | modular |
| New.CleanUp.ReferenceOTU41_PcA.KS_7883 | Stro2780_2 | 71 | 17 | 4.00E-26 | salinilactam | modular |
| New.CleanUp.ReferenceOTU42_PcA.KS_5408 | StiA_Q8RJY6_1KSB | 70 | 117 | 8.00E-43 | stigmatellin | KS1 |
| New.CleanUp.ReferenceOTU43_PcA.KS_5085 | SpnA_Q9ALM6_1KSB | 66 | 134 | 1.00E-46 | spinosad | modular |
| New.CleanUp.ReferenceOTU48_PcA.KS_6738 | TylGI_O33954_2mod | 71 | 134 | 6.00E-78 | tylosin | modular |
| New.CleanUp.ReferenceOTU48_PcA.KS_6738 | TylGI_O33954_2mod | 77 | 75 | 6.00E-78 | tylosin | modular |
| New.CleanUp.ReferenceOTU49_PcA.KS_7041 | EpoF_Q9L8C5_1mod | 78 | 59 | 9.00E-24 | epothilone | modular |
| New.CleanUp.ReferenceOTU50_PcA.KS_8029 | EpoE_Q9L8C6_1mod | 60 | 213 | 3.00E-66 | epothilone | modular |
| New.CleanUp.ReferenceOTU51_PcA.KS_6732 | JamE_AAS98777_KS1 | 59 | 126 | 4.00E-63 | jamaicamide | KS |
| New.CleanUp.ReferenceOTU51_PcA.KS_6732 | JamE_AAS98777_KS1 | 75 | 60 | 4.00E-63 | jamaicamide | KS |
| New.CleanUp.ReferenceOTU58_PcA.KS_7548 | StiE_Q8RJY2_1KSB | 58 | 142 | 1.00E-51 | stigmatellin | modular |
| New.CleanUp.ReferenceOTU58_PcA.KS_7548 | StiE_Q8RJY2_1KSB | 62 | 32 | 1.00E-51 | stigmatellin | modular |
| New.CleanUp.ReferenceOTU58_PcA.KS_7548 | StiE_Q8RJY2_1KSB | 67 | 21 | 1.00E-51 | stigmatellin | modular |
| New.CleanUp.ReferenceOTU6_PcA.KS_5303 | LipC_ABB05104_1KSB | 70 | 150 | 8.00E-60 | lipomycin | modular |
| New.CleanUp.ReferenceOTU61_PcA.KS_6415 | ChlB1_AAZ77673_i | 54 | 120 | 5.00E-46 | chlorothricin | iterative |
| New.CleanUp.ReferenceOTU61_PcA.KS_6415 | ChlB1_AAZ77673_i | 74 | 43 | 5.00E-46 | chlorothricin | iterative |
| New.CleanUp.ReferenceOTU61_PcA.KS_6415 | ChlB1_AAZ77673_i | 67 | 27 | 5.00E-46 | chlorothricin | iterative |
| New.CleanUp.ReferenceOTU62_PcA.KS_4285 | AveA3_Q9S0R4_3mod | 54 | 135 | 6.00E-49 | avermectin | modular |
| New.CleanUp.ReferenceOTU62_PcA.KS_4285 | AveA3_Q9S0R4_3mod | 65 | 54 | 6.00E-49 | avermectin | modular |
| New.CleanUp.ReferenceOTU62_PcA.KS_4285 | AveA3_Q9S0R4_3mod | 81 | 21 | 6.00E-49 | avermectin | modular |
| New.CleanUp.ReferenceOTU64_PcA.KS_6418 | NosB_Q9RAH3_H | 63 | 178 | 1.00E-61 | nostopeptolide | hybridKS |
| New.CleanUp.ReferenceOTU64_PcA.KS_6418 | NosB_Q9RAH3_H | 67 | 30 | 1.00E-61 | nostopeptolide | hybridKS |
| New.CleanUp.ReferenceOTU66_PcA.KS_6004 | JamK_AAS98782_mod | 67 | 220 | 2.00E-69 | jamaicamide | modular |
| New.CleanUp.ReferenceOTU67_PcA.KS_4944 | JamE_AAS98777_KS1 | 62 | 163 | 1.00E-57 | jamaicamide | KS |
| New.CleanUp.ReferenceOTU68_PcA.KS_6006 | CurI_AAT70104_mod | 59 | 192 | 8.00E-61 | curacin | modular |
| New.CleanUp.ReferenceOTU69_PcA.KS_8131 | JamL_AAS98783_mod | 58 | 158 | 2.00E-44 | jamaicamide | modular |
| New.CleanUp.ReferenceOTU69_PcA.KS_8131 | JamL_AAS98783_mod | 68 | 22 | 2.00E-44 | jamaicamide | modular |
| New.CleanUp.ReferenceOTU73_PcA.KS_7226 | ChlB1_AAZ77673_i | 53 | 152 | 3.00E-49 | chlorothricin | iterative |
| New.CleanUp.ReferenceOTU73_PcA.KS_7226 | ChlB1_AAZ77673_i | 57 | 35 | 3.00E-49 | chlorothricin | iterative |
| New.CleanUp.ReferenceOTU78_PcA.KS_4842 | VicC_BAD08359_1KSB | 72 | 65 | 2.00E-30 | vicenistatin | modular |
| New.CleanUp.ReferenceOTU78_PcA.KS_4842 | VicC_BAD08359_1KSB | 79 | 28 | 2.00E-30 | vicenistatin | modular |
| New.CleanUp.ReferenceOTU79_PcA.KS_4095 | ChlA2_AAZ77694_2KSB | 64 | 211 | 3.00E-72 | chlorothricin | modular |
| New.CleanUp.ReferenceOTU81_PcA.KS_3207 | JamK_AAS98782_mod | 64 | 123 | 6.00E-46 | jamaicamide | modular |
| New.CleanUp.ReferenceOTU81_PcA.KS_3207 | JamK_AAS98782_mod | 84 | 19 | 6.00E-46 | jamaicamide | modular |
| New.CleanUp.ReferenceOTU86_PcA.KS_5188 | PimS1_Q9X993_3KSB | 50 | 208 | 2.00E-46 | pimaricin | modular |
| New.CleanUp.ReferenceOTU90_PcA.KS_7319 | SpnD_Q9ALM3_3KSB | 67 | 98 | 5.00E-61 | spinosad | modular |
| New.CleanUp.ReferenceOTU90_PcA.KS_7319 | SpnD_Q9ALM3_3KSB | 61 | 87 | 5.00E-61 | spinosad | modular |
| New.CleanUp.ReferenceOTU92_PcA.KS_6863 | SpnD_Q9ALM3_1KSB | 69 | 213 | 3.00E-70 | spinosad | modular |
| New.CleanUp.ReferenceOTU95_PcA.KS_4447 | EpoC_Q9L8C8_H | 62 | 159 | 3.00E-61 | epothilone | hybridKS |
| New.CleanUp.ReferenceOTU95_PcA.KS_4447 | EpoC_Q9L8C8_H | 58 | 24 | 3.00E-61 | epothilone | hybridKS |
| New.CleanUp.ReferenceOTU95_PcA.KS_4447 | EpoC_Q9L8C8_H | 52 | 27 | 3.00E-61 | epothilone | hybridKS |
| New.CleanUp.ReferenceOTU96_PcA.KS_3644 | MxaB_Q93TX0_1KSB | 50 | 132 | 1.00E-51 | myxalamid | modular |
| New.CleanUp.ReferenceOTU96_PcA.KS_3644 | MxaB_Q93TX0_1KSB | 68 | 53 | 1.00E-51 | myxalamid | modular |
| New.CleanUp.ReferenceOTU96_PcA.KS_3644 | MxaB_Q93TX0_1KSB | 44 | 27 | 1.00E-51 | myxalamid | modular |
| New.CleanUp.ReferenceOTU100_SnC.KS_3847 | PikAII_Q9ZGI4_1KSB | 57 | 179 | 1.00E-49 | pikromycin | modular |
| New.CleanUp.ReferenceOTU115_SnC.KS_27131 | ChlA5_AAZ77698_1KSB | 64 | 203 | 7.00E-74 | chlorothricin | modular |
| New.CleanUp.ReferenceOTU118_SnA.KS_20033 | TetE_BAE93730_3mod | 74 | 76 | 3.00E-45 | tetronomycin | modular |
| New.CleanUp.ReferenceOTU118_SnA.KS_20033 | TetE_BAE93730_3mod | 61 | 77 | 3.00E-45 | tetronomycin | modular |
| New.CleanUp.ReferenceOTU118_SnA.KS_20033 | TetE_BAE93730_3mod | 73 | 15 | 3.00E-45 | tetronomycin | modular |
| New.CleanUp.ReferenceOTU118_SnA.KS_20033 | TetE_BAE93730_3mod | 75 | 12 | 3.00E-45 | tetronomycin | modular |
| New.CleanUp.ReferenceOTU123_SnC.KS_9934 | AveA2_Q9S0R7_3mod | 57 | 164 | 6.00E-50 | avermectin | modular |
| New.CleanUp.ReferenceOTU133_SnA.KS_26669 | CurI_AAT70104_mod | 59 | 179 | 5.00E-50 | curacin | modular |
| New.CleanUp.ReferenceOTU135_SnC.KS_6797 | SpnD_Q9ALM3_3KSB | 68 | 144 | 2.00E-55 | spinosad | modular |
| New.CleanUp.ReferenceOTU135_SnC.KS_6797 | SpnD_Q9ALM3_3KSB | 72 | 18 | 2.00E-55 | spinosad | modular |
| New.CleanUp.ReferenceOTU138_SnC.KS_32467 | JamM_AAS98784_H | 60 | 100 | 2.00E-56 | jamaicamide | hybridKS |
| New.CleanUp.ReferenceOTU138_SnC.KS_32467 | JamM_AAS98784_H | 67 | 64 | 2.00E-56 | jamaicamide | hybridKS |
| New.CleanUp.ReferenceOTU138_SnC.KS_32467 | JamM_AAS98784_H | 50 | 24 | 2.00E-56 | jamaicamide | hybridKS |
| New.CleanUp.ReferenceOTU144_SnA.KS_31766 | CurI_AAT70104_mod | 65 | 143 | 2.00E-52 | curacin | modular |
| New.CleanUp.ReferenceOTU149_SnA.KS_20234 | CALO5_12183629_i | 54 | 218 | 3.00E-60 | calicheamicin | iterative |
| New.CleanUp.ReferenceOTU161_SnC.KS_33548 | AveA4_Q9S0R3_2mod | 64 | 120 | 9.00E-42 | avermectin | modular |
| New.CleanUp.ReferenceOTU161_SnC.KS_33548 | AveA4_Q9S0R3_2mod | 85 | 13 | 9.00E-42 | avermectin | modular |
| New.CleanUp.ReferenceOTU165_SnC.KS_34237 | ChlB1_AAZ77673_i | 60 | 94 | 9.00E-47 | chlorothricin | iterative |
| New.CleanUp.ReferenceOTU165_SnC.KS_34237 | ChlB1_AAZ77673_i | 66 | 47 | 9.00E-47 | chlorothricin | iterative |
| New.CleanUp.ReferenceOTU165_SnC.KS_34237 | ChlB1_AAZ77673_i | 43 | 54 | 9.00E-47 | chlorothricin | iterative |
| New.CleanUp.ReferenceOTU166_SnC.KS_32005 | EpoD_Q9L8C7_4mod | 62 | 152 | 5.00E-63 | epothilone | modular |
| New.CleanUp.ReferenceOTU166_SnC.KS_32005 | EpoD_Q9L8C7_4mod | 64 | 47 | 5.00E-63 | epothilone | modular |
| New.CleanUp.ReferenceOTU178_SnC.KS_27407 | StiE_Q8RJY2_1KSB | 49 | 188 | 3.00E-40 | stigmatellin | modular |
| New.CleanUp.ReferenceOTU179_SnA.KS_20095 | JamK_AAS98782_mod | 52 | 183 | 8.00E-47 | jamaicamide | modular |
| New.CleanUp.ReferenceOTU179_SnA.KS_20095 | JamK_AAS98782_mod | 89 | 9 | 8.00E-47 | jamaicamide | modular |
| New.CleanUp.ReferenceOTU180_SnC.KS_23940 | FurC1_ABB88521_KSB | 67 | 90 | 6.00E-37 | 5-alkenyl-3,3(2h)-furanone | modular |
| New.CleanUp.ReferenceOTU180_SnC.KS_23940 | FurC1_ABB88521_KSB | 83 | 23 | 6.00E-37 | 5-alkenyl-3,3(2h)-furanone | modular |
| New.CleanUp.ReferenceOTU203_SnC.KS_18676 | StiE_Q8RJY2_1KSB | 50 | 210 | 3.00E-52 | stigmatellin | modular |
| New.CleanUp.ReferenceOTU204_SnC.KS_22809 | EcoE_AAX98188_3KSB | 65 | 195 | 4.00E-73 | eco-02301 | modular |
| New.CleanUp.ReferenceOTU204_SnC.KS_22809 | EcoE_AAX98188_3KSB | 57 | 23 | 4.00E-73 | eco-02301 | modular |
| New.CleanUp.ReferenceOTU217_SnC.KS_13158 | JamE_AAS98777_KS1 | 58 | 95 | 8.00E-28 | jamaicamide | KS |
| New.CleanUp.ReferenceOTU219_SnC.KS_31427 | AveA4_Q9S0R3_2mod | 51 | 156 | 1.00E-33 | avermectin | modular |
| New.CleanUp.ReferenceOTU221_SnA.KS_26896 | JamE_AAS98777_KS1 | 57 | 191 | 7.00E-58 | jamaicamide | KS |
| New.CleanUp.ReferenceOTU221_SnA.KS_26896 | JamE_AAS98777_KS1 | 72 | 18 | 7.00E-58 | jamaicamide | KS |
| New.CleanUp.ReferenceOTU223_SnC.KS_28590 | LipC_ABB05104_1KSB | 68 | 115 | 5.00E-58 | lipomycin | modular |
| New.CleanUp.ReferenceOTU223_SnC.KS_28590 | LipC_ABB05104_1KSB | 63 | 60 | 5.00E-58 | lipomycin | modular |
| New.CleanUp.ReferenceOTU225_SnC.KS_31358 | CurJ_AAT70105_mod | 60 | 155 | 2.00E-60 | curacin | modular |
| New.CleanUp.ReferenceOTU225_SnC.KS_31358 | CurJ_AAT70105_mod | 59 | 61 | 2.00E-60 | curacin | modular |
| New.CleanUp.ReferenceOTU230_SnC.KS_17755 | JamE_AAS98777_KS1 | 52 | 203 | 3.00E-59 | jamaicamide | KS |
| New.CleanUp.ReferenceOTU230_SnC.KS_17755 | JamE_AAS98777_KS1 | 55 | 20 | 3.00E-59 | jamaicamide | KS |
| New.CleanUp.ReferenceOTU232_SnA.KS_34475 | TylGIII_O33956_1mod | 53 | 188 | 2.00E-46 | tylosin | modular |
| New.CleanUp.ReferenceOTU232_SnA.KS_34475 | TylGIII_O33956_1mod | 82 | 33 | 2.00E-46 | tylosin | modular |
| New.CleanUp.ReferenceOTU235_SnC.KS_14184 | StiG_Q8RJY0_1KSB | 72 | 87 | 7.00E-38 | stigmatellin | modular |
| New.CleanUp.ReferenceOTU235_SnC.KS_14184 | StiG_Q8RJY0_1KSB | 71 | 21 | 7.00E-38 | stigmatellin | modular |
| New.CleanUp.ReferenceOTU236_SnC.KS_8939 | EpoD_Q9L8C7_4mod | 55 | 230 | 9.00E-65 | epothilone | modular |
| New.CleanUp.ReferenceOTU239_SnC.KS_25668 | CurA_AAT70096_mod | 69 | 68 | 3.00E-42 | curacin | KS |
| New.CleanUp.ReferenceOTU239_SnC.KS_25668 | CurA_AAT70096_mod | 52 | 86 | 3.00E-42 | curacin | KS |
| New.CleanUp.ReferenceOTU239_SnC.KS_25668 | CurA_AAT70096_mod | 69 | 16 | 3.00E-42 | curacin | KS |
| New.CleanUp.ReferenceOTU240_SnC.KS_23492 | AveA4_Q9S0R3_2mod | 76 | 137 | 2.00E-62 | avermectin | modular |
| New.CleanUp.ReferenceOTU240_SnC.KS_23492 | AveA4_Q9S0R3_2mod | 68 | 28 | 2.00E-62 | avermectin | modular |
| New.CleanUp.ReferenceOTU246_SnC.KS_28319 | SpnA_Q9ALM6_1KSB | 65 | 134 | 3.00E-45 | spinosad | modular |
| New.CleanUp.ReferenceOTU254_SnA.KS_34590 | ChlB1_AAZ77673_i | 51 | 173 | 3.00E-45 | chlorothricin | iterative |
| New.CleanUp.ReferenceOTU254_SnA.KS_34590 | ChlB1_AAZ77673_i | 56 | 36 | 3.00E-45 | chlorothricin | iterative |
| New.CleanUp.ReferenceOTU254_SnA.KS_34590 | ChlB1_AAZ77673_i | 73 | 11 | 3.00E-45 | chlorothricin | iterative |
| New.CleanUp.ReferenceOTU257_SnA.KS_35444 | LnmJ_AF484556_4T | 56 | 63 | 2.00E-25 | leinamycin | trans |
| New.CleanUp.ReferenceOTU257_SnA.KS_35444 | LnmJ_AF484556_4T | 51 | 37 | 2.00E-25 | leinamycin | trans |
| New.CleanUp.ReferenceOTU257_SnA.KS_35444 | LnmJ_AF484556_4T | 71 | 17 | 2.00E-25 | leinamycin | trans |
| New.CleanUp.ReferenceOTU257_SnA.KS_35444 | LnmJ_AF484556_4T | 38 | 32 | 2.00E-25 | leinamycin | trans |
| New.CleanUp.ReferenceOTU258_SnC.KS_36616 | StiF_Q8RJY1_1KSB | 53 | 92 | 1.00E-24 | stigmatellin | modular |
| New.CleanUp.ReferenceOTU262_SnA.KS_21832 | CALO5_12183629_i | 64 | 81 | 6.00E-27 | calicheamicin | iterative |
| New.CleanUp.ReferenceOTU262_SnA.KS_21832 | CALO5_12183629_i | 69 | 13 | 6.00E-27 | calicheamicin | iterative |
| New.CleanUp.ReferenceOTU264_SnA.KS_20245 | JamE_AAS98777_KS1 | 63 | 84 | 6.00E-21 | jamaicamide | KS |
| New.CleanUp.ReferenceOTU266_SnC.KS_29268 | ChlB1_AAZ77673_i | 61 | 180 | 2.00E-59 | chlorothricin | iterative |
| New.CleanUp.ReferenceOTU271_SnC.KS_7918 | AveA4_Q9S0R3_2mod | 60 | 108 | 3.00E-39 | avermectin | modular |
| New.CleanUp.ReferenceOTU271_SnC.KS_7918 | AveA4_Q9S0R3_2mod | 86 | 21 | 3.00E-39 | avermectin | modular |
| New.CleanUp.ReferenceOTU272_SnC.KS_11826 | EpoD_Q9L8C7_4mod | 62 | 133 | 2.00E-48 | epothilone | modular |
| New.CleanUp.ReferenceOTU275_SnA.KS_12135 | MtaB_Q9RFL0_1KSB | 56 | 109 | 3.00E-49 | myxothiazol | KS1 |
| New.CleanUp.ReferenceOTU275_SnA.KS_12135 | MtaB_Q9RFL0_1KSB | 56 | 48 | 3.00E-49 | myxothiazol | KS1 |
| New.CleanUp.ReferenceOTU275_SnA.KS_12135 | MtaB_Q9RFL0_1KSB | 38 | 65 | 3.00E-49 | myxothiazol | KS1 |
| New.CleanUp.ReferenceOTU276_SnC.KS_25657 | JamE_AAS98777_KS1 | 42 | 161 | 4.00E-35 | jamaicamide | KS |
| New.CleanUp.ReferenceOTU276_SnC.KS_25657 | JamE_AAS98777_KS1 | 58 | 33 | 4.00E-35 | jamaicamide | KS |
| New.CleanUp.ReferenceOTU277_SnC.KS_16211 | AveA4_Q9S0R3_3mod | 65 | 213 | 2.00E-73 | avermectin | modular |
| New.CleanUp.ReferenceOTU278_SnC.KS_36608 | AveA4_Q9S0R3_2mod | 46 | 194 | 3.00E-33 | avermectin | modular |
| New.CleanUp.ReferenceOTU281_SnC.KS_19700 | AveA2_Q9S0R7_4mod | 79 | 150 | 2.00E-58 | avermectin | modular |
| New.CleanUp.ReferenceOTU30_SnA.KS_23211 | StiG_Q8RJY0_1KSB | 66 | 106 | 2.00E-59 | stigmatellin | modular |
| New.CleanUp.ReferenceOTU30_SnA.KS_23211 | StiG_Q8RJY0_1KSB | 57 | 84 | 2.00E-59 | stigmatellin | modular |
| New.CleanUp.ReferenceOTU300_SnC.KS_12556 | AveA4_Q9S0R3_2mod | 82 | 57 | 8.00E-34 | avermectin | modular |
| New.CleanUp.ReferenceOTU300_SnC.KS_12556 | AveA4_Q9S0R3_2mod | 71 | 35 | 8.00E-34 | avermectin | modular |
| New.CleanUp.ReferenceOTU38_SnC.KS_26004 | EpoD_Q9L8C7_4mod | 66 | 168 | 3.00E-63 | epothilone | modular |
| New.CleanUp.ReferenceOTU47_SnC.KS_9398 | JamE_AAS98777_KS1 | 51 | 165 | 2.00E-47 | jamaicamide | KS |
| New.CleanUp.ReferenceOTU48_SnC.KS_36821 | CurL_AAT70107_mod | 56 | 148 | 8.00E-53 | curacin | modular |
| New.CleanUp.ReferenceOTU48_SnC.KS_36821 | CurL_AAT70107_mod | 57 | 49 | 8.00E-53 | curacin | modular |
| New.CleanUp.ReferenceOTU51_SnC.KS_16295 | JamE_AAS98777_KS1 | 57 | 149 | 5.00E-61 | jamaicamide | KS |
| New.CleanUp.ReferenceOTU51_SnC.KS_16295 | JamE_AAS98777_KS1 | 49 | 63 | 5.00E-61 | jamaicamide | KS |
| New.CleanUp.ReferenceOTU55_SnC.KS_22107 | StiG_Q8RJY0_1KSB | 57 | 157 | 4.00E-75 | stigmatellin | modular |
| New.CleanUp.ReferenceOTU55_SnC.KS_22107 | StiG_Q8RJY0_1KSB | 75 | 67 | 4.00E-75 | stigmatellin | modular |
| New.CleanUp.ReferenceOTU57_SnC.KS_26481 | Stro2778_1 | 67 | 89 | 1.00E-58 | salinilactam | modular |
| New.CleanUp.ReferenceOTU57_SnC.KS_26481 | Stro2778_1 | 52 | 106 | 1.00E-58 | salinilactam | modular |
| New.CleanUp.ReferenceOTU57_SnC.KS_26481 | Stro2778_1 | 71 | 17 | 1.00E-58 | salinilactam | modular |
| New.CleanUp.ReferenceOTU65_SnC.KS_28498 | ChlB1_AAZ77673_i | 60 | 181 | 4.00E-69 | chlorothricin | iterative |
| New.CleanUp.ReferenceOTU65_SnC.KS_28498 | ChlB1_AAZ77673_i | 62 | 45 | 4.00E-69 | chlorothricin | iterative |
| New.CleanUp.ReferenceOTU88_SnC.KS_31623 | EpoD_Q9L8C7_4mod | 60 | 143 | 2.00E-59 | epothilone | modular |
| New.CleanUp.ReferenceOTU88_SnC.KS_31623 | EpoD_Q9L8C7_4mod | 58 | 57 | 2.00E-59 | epothilone | modular |
| New.CleanUp.ReferenceOTU90_SnC.KS_12662 | MtaD_Q9RFK8_1KSB | 51 | 175 | 1.00E-52 | myxothiazol | hybridKS |
| New.CleanUp.ReferenceOTU90_SnC.KS_12662 | MtaD_Q9RFK8_1KSB | 88 | 16 | 1.00E-52 | myxothiazol | hybridKS |
| New.CleanUp.ReferenceOTU92_SnC.KS_17813 | AveA4_Q9S0R3_2mod | 56 | 171 | 5.00E-60 | avermectin | modular |
| New.CleanUp.ReferenceOTU92_SnC.KS_17813 | AveA4_Q9S0R3_2mod | 71 | 45 | 5.00E-60 | avermectin | modular |
| New.CleanUp.ReferenceOTU99_SnC.KS_31079 | SpnC_Q9ALM4_2KSB | 60 | 142 | 4.00E-55 | spinosad | modular |
| New.CleanUp.ReferenceOTU99_SnC.KS_31079 | SpnC_Q9ALM4_2KSB | 68 | 34 | 4.00E-55 | spinosad | modular |
| New.CleanUp.ReferenceOTU101_IpB.KS_8118 | StiE_Q8RJY2_1KSB | 59 | 129 | 1.00E-58 | stigmatellin | modular |
| New.CleanUp.ReferenceOTU101_IpB.KS_8118 | StiE_Q8RJY2_1KSB | 68 | 60 | 1.00E-58 | stigmatellin | modular |
| New.CleanUp.ReferenceOTU108_IpB.KS_6810 | EcoA_AAX98184_2KSB | 82 | 28 | 2.00E-17 | eco-02301 | modular |
| New.CleanUp.ReferenceOTU108_IpB.KS_6810 | EcoA_AAX98184_2KSB | 53 | 32 | 2.00E-17 | eco-02301 | modular |
| New.CleanUp.ReferenceOTU108_IpB.KS_6810 | EcoA_AAX98184_2KSB | 50 | 20 | 2.00E-17 | eco-02301 | modular |
| New.CleanUp.ReferenceOTU108_IpB.KS_6810 | EcoA_AAX98184_2KSB | 59 | 17 | 2.00E-17 | eco-02301 | modular |
| New.CleanUp.ReferenceOTU111_IpB.KS_7399 | LnmJ_AF484556_2T | 54 | 79 | 1.00E-36 | leinamycin | trans |
| New.CleanUp.ReferenceOTU111_IpB.KS_7399 | LnmJ_AF484556_2T | 44 | 77 | 1.00E-36 | leinamycin | trans |
| New.CleanUp.ReferenceOTU111_IpB.KS_7399 | LnmJ_AF484556_2T | 71 | 17 | 1.00E-36 | leinamycin | trans |
| New.CleanUp.ReferenceOTU112_IpB.KS_4072 | SpnD_Q9ALM3_3KSB | 70 | 93 | 1.00E-61 | spinosad | modular |
| New.CleanUp.ReferenceOTU112_IpB.KS_4072 | SpnD_Q9ALM3_3KSB | 51 | 117 | 1.00E-61 | spinosad | modular |
| New.CleanUp.ReferenceOTU113_IpB.KS_5760 | CurJ_AAT70105_mod | 54 | 167 | 2.00E-35 | curacin | modular |
| New.CleanUp.ReferenceOTU115_IpB.KS_6919 | EpoE_Q9L8C6_1mod | 58 | 160 | 4.00E-42 | epothilone | modular |
| New.CleanUp.ReferenceOTU115_IpB.KS_6919 | EpoE_Q9L8C6_1mod | 48 | 23 | 4.00E-42 | epothilone | modular |
| New.CleanUp.ReferenceOTU120_IpB.KS_7317 | MxaB_Q93TX0_1KSB | 58 | 162 | 6.00E-61 | myxalamid | modular |
| New.CleanUp.ReferenceOTU120_IpB.KS_7317 | MxaB_Q93TX0_1KSB | 65 | 43 | 6.00E-61 | myxalamid | modular |
| New.CleanUp.ReferenceOTU120_IpB.KS_7317 | MxaB_Q93TX0_1KSB | 73 | 11 | 6.00E-61 | myxalamid | modular |
| New.CleanUp.ReferenceOTU122_IpB.KS_3399 | Sare1246_1 | 63 | 107 | 1.00E-45 | rifamycin | modular |
| New.CleanUp.ReferenceOTU122_IpB.KS_3399 | Sare1246_1 | 78 | 36 | 1.00E-45 | rifamycin | modular |
| New.CleanUp.ReferenceOTU125_IpB.KS_5475 | MtaE_Q9RFK7_1KSB | 59 | 209 | 3.00E-68 | myxothiazol | modular |
| New.CleanUp.ReferenceOTU133_IpB.KS_10209 | StiG_Q8RJY0_1KSB | 60 | 195 | 6.00E-61 | stigmatellin | modular |
| New.CleanUp.ReferenceOTU136_IpB.KS_8219 | StiG_Q8RJY0_1KSB | 51 | 196 | 1.00E-42 | stigmatellin | modular |
| New.CleanUp.ReferenceOTU14_IpB.KS_9834 | StiE_Q8RJY2_1KSB | 50 | 110 | 3.00E-37 | stigmatellin | modular |
| New.CleanUp.ReferenceOTU14_IpB.KS_9834 | StiE_Q8RJY2_1KSB | 58 | 52 | 3.00E-37 | stigmatellin | modular |
| New.CleanUp.ReferenceOTU14_IpB.KS_9834 | StiE_Q8RJY2_1KSB | 42 | 24 | 3.00E-37 | stigmatellin | modular |
| New.CleanUp.ReferenceOTU141_IpB.KS_4989 | CurM_AAT70108_mod | 75 | 122 | 4.00E-63 | curacin | modular |
| New.CleanUp.ReferenceOTU141_IpB.KS_4989 | CurM_AAT70108_mod | 47 | 74 | 4.00E-63 | curacin | modular |
| New.CleanUp.ReferenceOTU15_IpB.KS_7033 | CurI_AAT70104_mod | 63 | 146 | 5.00E-53 | curacin | modular |
| New.CleanUp.ReferenceOTU15_IpB.KS_7033 | CurI_AAT70104_mod | 44 | 41 | 5.00E-53 | curacin | modular |
| New.CleanUp.ReferenceOTU151_IpB.KS_3662 | CALO5_12183629_i | 50 | 217 | 1.00E-53 | calicheamicin | iterative |
| New.CleanUp.ReferenceOTU154_IpB.KS_8928 | NosB_Q9RAH3_H | 73 | 123 | 4.00E-75 | nostopeptolide | hybridKS |
| New.CleanUp.ReferenceOTU154_IpB.KS_8928 | NosB_Q9RAH3_H | 58 | 96 | 4.00E-75 | nostopeptolide | hybridKS |
| New.CleanUp.ReferenceOTU155_IpB.KS_5402 | EpoD_Q9L8C7_3mod | 56 | 115 | 7.00E-55 | epothilone | modular |
| New.CleanUp.ReferenceOTU155_IpB.KS_5402 | EpoD_Q9L8C7_3mod | 69 | 77 | 7.00E-55 | epothilone | modular |
| New.CleanUp.ReferenceOTU164_IpB.KS_8206 | JamL_AAS98783_mod | 59 | 180 | 4.00E-46 | jamaicamide | modular |
| New.CleanUp.ReferenceOTU168_IpB.KS_4544 | MxaC_Q93TW9_3KSB | 55 | 110 | 2.00E-33 | myxalamid | modular |
| New.CleanUp.ReferenceOTU170_IpB.KS_6099 | StiB_Q8RJY5_1KSB | 50 | 105 | 2.00E-49 | stigmatellin | modular |
| New.CleanUp.ReferenceOTU170_IpB.KS_6099 | StiB_Q8RJY5_1KSB | 57 | 81 | 2.00E-49 | stigmatellin | modular |
| New.CleanUp.ReferenceOTU176_IpB.KS_8873 | JamK_AAS98782_mod | 50 | 216 | 2.00E-52 | jamaicamide | modular |
| New.CleanUp.ReferenceOTU177_IpB.KS_5623 | StiG_Q8RJY0_1KSB | 59 | 195 | 1.00E-53 | stigmatellin | modular |
| New.CleanUp.ReferenceOTU177_IpB.KS_5623 | StiG_Q8RJY0_1KSB | 73 | 15 | 1.00E-53 | stigmatellin | modular |
| New.CleanUp.ReferenceOTU181_IpB.KS_2713 | SpnC_Q9ALM4_2KSB | 61 | 49 | 1.00E-32 | spinosad | modular |
| New.CleanUp.ReferenceOTU181_IpB.KS_2713 | SpnC_Q9ALM4_2KSB | 74 | 34 | 1.00E-32 | spinosad | modular |
| New.CleanUp.ReferenceOTU181_IpB.KS_2713 | SpnC_Q9ALM4_2KSB | 64 | 36 | 1.00E-32 | spinosad | modular |
| New.CleanUp.ReferenceOTU183_IpB.KS_8569 | CurJ_AAT70105_mod | 48 | 196 | 3.00E-33 | curacin | modular |
| New.CleanUp.ReferenceOTU184_IpB.KS_8894 | CurK_AAT70106_mod | 64 | 112 | 6.00E-44 | curacin | modular |
| New.CleanUp.ReferenceOTU184_IpB.KS_8894 | CurK_AAT70106_mod | 37 | 52 | 6.00E-44 | curacin | modular |
| New.CleanUp.ReferenceOTU186_IpB.KS_6682 | JamK_AAS98782_mod | 67 | 127 | 1.00E-60 | jamaicamide | modular |
| New.CleanUp.ReferenceOTU186_IpB.KS_6682 | JamK_AAS98782_mod | 62 | 86 | 1.00E-60 | jamaicamide | modular |
| New.CleanUp.ReferenceOTU187_IpB.KS_7576 | CurI_AAT70104_mod | 71 | 89 | 1.00E-53 | curacin | modular |
| New.CleanUp.ReferenceOTU187_IpB.KS_7576 | CurI_AAT70104_mod | 69 | 39 | 1.00E-53 | curacin | modular |
| New.CleanUp.ReferenceOTU187_IpB.KS_7576 | CurI_AAT70104_mod | 48 | 61 | 1.00E-53 | curacin | modular |
| New.CleanUp.ReferenceOTU187_IpB.KS_7576 | CurI_AAT70104_mod | 50 | 18 | 1.00E-53 | curacin | modular |
| New.CleanUp.ReferenceOTU188_IpB.KS_7041 | JamJ_AAS98781 | 51 | 108 | 2.00E-53 | jamaicamide | modular |
| New.CleanUp.ReferenceOTU188_IpB.KS_7041 | JamJ_AAS98781 | 49 | 73 | 2.00E-53 | jamaicamide | modular |
| New.CleanUp.ReferenceOTU188_IpB.KS_7041 | JamJ_AAS98781 | 62 | 40 | 2.00E-53 | jamaicamide | modular |
| New.CleanUp.ReferenceOTU191_IpB.KS_7271 | LipA_ABB05102_1KSB | 54 | 71 | 1.00E-12 | lipomycin | modular |
| New.CleanUp.ReferenceOTU20_IpB.KS_9626 | FurD2_ABB88522_KSB | 59 | 169 | 2.00E-55 | 5-alkenyl-3,3(2h)-furanone | modular |
| New.CleanUp.ReferenceOTU204_IpB.KS_7291 | CurI_AAT70104_mod | 71 | 85 | 4.00E-32 | curacin | modular |
| New.CleanUp.ReferenceOTU205_IpB.KS_7293 | EpoE_Q9L8C6_1mod | 49 | 94 | 3.00E-22 | epothilone | modular |
| New.CleanUp.ReferenceOTU205_IpB.KS_7293 | EpoE_Q9L8C6_1mod | 75 | 16 | 3.00E-22 | epothilone | modular |
| New.CleanUp.ReferenceOTU205_IpB.KS_7293 | EpoE_Q9L8C6_1mod | 36 | 191 | 2.00E-18 | epothilone | modular |
| New.CleanUp.ReferenceOTU206_IpB.KS_5133 | StiA_Q8RJY6_1KSB | 65 | 207 | 2.00E-71 | stigmatellin | KS1 |
| New.CleanUp.ReferenceOTU211_IpB.KS_6536 | CurA_AAT70096_mod | 68 | 209 | 2.00E-69 | curacin | KS |
| New.CleanUp.ReferenceOTU211_IpB.KS_6536 | CurA_AAT70096_mod | 73 | 11 | 2.00E-69 | curacin | KS |
| New.CleanUp.ReferenceOTU213_IpB.KS_2471 | PimS2_Q9EWA1_2KSB | 62 | 199 | 2.00E-65 | pimaricin | modular |
| New.CleanUp.ReferenceOTU215_IpB.KS_788 | JamM_AAS98784_H | 73 | 41 | 7.00E-14 | jamaicamide | hybridKS |
| New.CleanUp.ReferenceOTU223_IpB.KS_9401 | StiG_Q8RJY0_1KSB | 66 | 121 | 1.00E-57 | stigmatellin | modular |
| New.CleanUp.ReferenceOTU223_IpB.KS_9401 | StiG_Q8RJY0_1KSB | 59 | 54 | 1.00E-57 | stigmatellin | modular |
| New.CleanUp.ReferenceOTU23_IpB.KS_8804 | StiI_Q8RJX8_1KSB | 44 | 224 | 4.00E-39 | stigmatellin | modular |
| New.CleanUp.ReferenceOTU230_IpB.KS_8268 | MxaD_Q93TW8_1KSB | 60 | 127 | 9.00E-39 | myxalamid | modular |
| New.CleanUp.ReferenceOTU230_IpB.KS_8268 | MxaD_Q93TW8_1KSB | 91 | 11 | 9.00E-39 | myxalamid | modular |
| New.CleanUp.ReferenceOTU234_IpB.KS_4135 | JamL_AAS98783_mod | 66 | 80 | 7.00E-61 | jamaicamide | modular |
| New.CleanUp.ReferenceOTU234_IpB.KS_4135 | JamL_AAS98783_mod | 64 | 67 | 7.00E-61 | jamaicamide | modular |
| New.CleanUp.ReferenceOTU234_IpB.KS_4135 | JamL_AAS98783_mod | 40 | 63 | 7.00E-61 | jamaicamide | modular |
| New.CleanUp.ReferenceOTU234_IpB.KS_4135 | JamL_AAS98783_mod | 82 | 22 | 7.00E-61 | jamaicamide | modular |
| New.CleanUp.ReferenceOTU237_IpB.KS_5662 | CALO5_12183629_i | 55 | 196 | 4.00E-67 | calicheamicin | iterative |
| New.CleanUp.ReferenceOTU237_IpB.KS_5662 | CALO5_12183629_i | 71 | 31 | 4.00E-67 | calicheamicin | iterative |
| New.CleanUp.ReferenceOTU239_IpB.KS_7627 | MxaB_Q93TX0_1KSB | 67 | 144 | 1.00E-61 | myxalamid | modular |
| New.CleanUp.ReferenceOTU239_IpB.KS_7627 | MxaB_Q93TX0_1KSB | 55 | 38 | 1.00E-61 | myxalamid | modular |
| New.CleanUp.ReferenceOTU239_IpB.KS_7627 | MxaB_Q93TX0_1KSB | 57 | 21 | 1.00E-61 | myxalamid | modular |
| New.CleanUp.ReferenceOTU24_IpB.KS_9898 | AveA2_Q9S0R7_4mod | 69 | 157 | 1.00E-57 | avermectin | modular |
| New.CleanUp.ReferenceOTU241_IpB.KS_8150 | EcoA_AAX98184_2KSB | 49 | 220 | 2.00E-48 | eco-02301 | modular |
| New.CleanUp.ReferenceOTU243_IpB.KS_7133 | JamK_AAS98782_mod | 49 | 72 | 1.00E-43 | jamaicamide | modular |
| New.CleanUp.ReferenceOTU243_IpB.KS_7133 | JamK_AAS98782_mod | 65 | 46 | 1.00E-43 | jamaicamide | modular |
| New.CleanUp.ReferenceOTU243_IpB.KS_7133 | JamK_AAS98782_mod | 58 | 57 | 1.00E-43 | jamaicamide | modular |
| New.CleanUp.ReferenceOTU248_IpB.KS_3755 | EpoE_Q9L8C6_1mod | 64 | 112 | 3.00E-58 | epothilone | modular |
| New.CleanUp.ReferenceOTU248_IpB.KS_3755 | EpoE_Q9L8C6_1mod | 57 | 49 | 3.00E-58 | epothilone | modular |
| New.CleanUp.ReferenceOTU248_IpB.KS_3755 | EpoE_Q9L8C6_1mod | 52 | 46 | 3.00E-58 | epothilone | modular |
| New.CleanUp.ReferenceOTU251_IpB.KS_9798 | CurI_AAT70104_mod | 59 | 161 | 4.00E-55 | curacin | modular |
| New.CleanUp.ReferenceOTU251_IpB.KS_9798 | CurI_AAT70104_mod | 73 | 30 | 4.00E-55 | curacin | modular |
| New.CleanUp.ReferenceOTU254_IpB.KS_5431 | StiC_Q8RJY4_1KSB | 64 | 173 | 2.00E-56 | stigmatellin | modular |
| New.CleanUp.ReferenceOTU258_IpB.KS_8813 | MxaB_Q93TX0_1KSB | 69 | 80 | 3.00E-62 | myxalamid | modular |
| New.CleanUp.ReferenceOTU258_IpB.KS_8813 | MxaB_Q93TX0_1KSB | 61 | 90 | 3.00E-62 | myxalamid | modular |
| New.CleanUp.ReferenceOTU258_IpB.KS_8813 | MxaB_Q93TX0_1KSB | 43 | 46 | 3.00E-62 | myxalamid | modular |
| New.CleanUp.ReferenceOTU259_IpB.KS_9796 | ChlB1_AAZ77673_i | 65 | 139 | 6.00E-60 | chlorothricin | iterative |
| New.CleanUp.ReferenceOTU259_IpB.KS_9796 | ChlB1_AAZ77673_i | 66 | 41 | 6.00E-60 | chlorothricin | iterative |
| New.CleanUp.ReferenceOTU27_IpB.KS_5488 | LnmJ_AF484556_2T | 47 | 135 | 1.00E-31 | leinamycin | trans |
| New.CleanUp.ReferenceOTU28_IpB.KS_8803 | LnmI_AF484556_2T | 64 | 108 | 4.00E-29 | leinamycin | trans |
| New.CleanUp.ReferenceOTU28_IpB.KS_8803 | LnmI_AF484556_2T | 64 | 14 | 4.00E-29 | leinamycin | trans |
| New.CleanUp.ReferenceOTU38_IpB.KS_9035 | LipD_ABB05105_2KSB | 61 | 71 | 1.00E-35 | lipomycin | modular |
| New.CleanUp.ReferenceOTU38_IpB.KS_9035 | LipD_ABB05105_2KSB | 79 | 58 | 1.00E-35 | lipomycin | modular |
| New.CleanUp.ReferenceOTU4_IpB.KS_8418 | EpoC_Q9L8C8_H | 64 | 141 | 1.00E-47 | epothilone | hybridKS |
| New.CleanUp.ReferenceOTU45_IpB.KS_5599 | Sare1250_2 | 51 | 102 | 4.00E-23 | rifamycin | modular |
| New.CleanUp.ReferenceOTU46_IpB.KS_5452 | EpoD_Q9L8C7_4mod | 66 | 219 | 7.00E-81 | epothilone | modular |
| New.CleanUp.ReferenceOTU47_IpB.KS_9400 | EpoD_Q9L8C7_4mod | 69 | 48 | 3.00E-16 | epothilone | modular |
| New.CleanUp.ReferenceOTU48_IpB.KS_5455 | JamE_AAS98777_KS1 | 57 | 142 | 1.00E-61 | jamaicamide | KS |
| New.CleanUp.ReferenceOTU48_IpB.KS_5455 | JamE_AAS98777_KS1 | 61 | 62 | 1.00E-61 | jamaicamide | KS |
| New.CleanUp.ReferenceOTU5_IpB.KS_9568 | NosB_Q9RAH3_H | 51 | 193 | 1.00E-48 | nostopeptolide | hybridKS |
| New.CleanUp.ReferenceOTU52_IpB.KS_6840 | StiE_Q8RJY2_1KSB | 50 | 181 | 1.00E-46 | stigmatellin | modular |
| New.CleanUp.ReferenceOTU52_IpB.KS_6840 | StiE_Q8RJY2_1KSB | 55 | 29 | 1.00E-46 | stigmatellin | modular |
| New.CleanUp.ReferenceOTU6_IpB.KS_4876 | EpoD_Q9L8C7_4mod | 66 | 192 | 2.00E-69 | epothilone | modular |
| New.CleanUp.ReferenceOTU62_IpB.KS_9351 | JamE_AAS98777_KS1 | 47 | 225 | 7.00E-46 | jamaicamide | KS |
| New.CleanUp.ReferenceOTU70_IpB.KS_7847 | ChlB1_AAZ77673_i | 56 | 153 | 2.00E-49 | chlorothricin | iterative |
| New.CleanUp.ReferenceOTU70_IpB.KS_7847 | ChlB1_AAZ77673_i | 77 | 35 | 2.00E-49 | chlorothricin | iterative |
| New.CleanUp.ReferenceOTU71_IpB.KS_8182 | StiC_Q8RJY4_1KSB | 69 | 144 | 4.00E-57 | stigmatellin | modular |
| New.CleanUp.ReferenceOTU71_IpB.KS_8182 | StiC_Q8RJY4_1KSB | 77 | 13 | 4.00E-57 | stigmatellin | modular |
| New.CleanUp.ReferenceOTU78_IpB.KS_8907 | StiG_Q8RJY0_1KSB | 46 | 151 | 4.00E-30 | stigmatellin | modular |
| New.CleanUp.ReferenceOTU78_IpB.KS_8907 | StiG_Q8RJY0_1KSB | 39 | 150 | 1.00E-19 | stigmatellin | modular |
| New.CleanUp.ReferenceOTU79_IpB.KS_5779 | StiD_Q8RJY3_1KSB | 51 | 126 | 7.00E-35 | stigmatellin | modular |
| New.CleanUp.ReferenceOTU79_IpB.KS_5779 | StiD_Q8RJY3_1KSB | 60 | 42 | 7.00E-35 | stigmatellin | modular |
| New.CleanUp.ReferenceOTU79_IpB.KS_5779 | StiD_Q8RJY3_1KSB | 38 | 130 | 5.00E-15 | stigmatellin | modular |
| New.CleanUp.ReferenceOTU81_IpB.KS_5588 | MxaF_Q93TW6_1KSB | 66 | 82 | 3.00E-41 | myxalamid | KS1 |
| New.CleanUp.ReferenceOTU81_IpB.KS_5588 | MxaF_Q93TW6_1KSB | 71 | 62 | 3.00E-41 | myxalamid | KS1 |
| New.CleanUp.ReferenceOTU86_IpB.KS_6372 | CurI_AAT70104_mod | 69 | 189 | 7.00E-65 | curacin | modular |
| New.CleanUp.ReferenceOTU89_IpB.KS_7279 | EpoD_Q9L8C7_4mod | 73 | 164 | 1.00E-70 | epothilone | modular |
| New.CleanUp.ReferenceOTU89_IpB.KS_7279 | EpoD_Q9L8C7_4mod | 70 | 20 | 1.00E-70 | epothilone | modular |
| New.CleanUp.ReferenceOTU92_IpB.KS_5346 | StiG_Q8RJY0_1KSB | 45 | 220 | 3.00E-43 | stigmatellin | modular |
| New.CleanUp.ReferenceOTU97_IpB.KS_3095 | CurA_AAT70096_mod | 60 | 93 | 6.00E-52 | curacin | KS |
| New.CleanUp.ReferenceOTU97_IpB.KS_3095 | CurA_AAT70096_mod | 64 | 45 | 6.00E-52 | curacin | KS |
| New.CleanUp.ReferenceOTU97_IpB.KS_3095 | CurA_AAT70096_mod | 63 | 41 | 6.00E-52 | curacin | KS |
| New.CleanUp.ReferenceOTU97_IpB.KS_3095 | CurA_AAT70096_mod | 67 | 27 | 6.00E-52 | curacin | KS |
